# Quorum quenching by endophytic *Bacillus cereus* AL1: a lactonase-based anti-virulence strategy against *Pseudomonas aeruginosa*

**DOI:** 10.1186/s12866-025-04396-4

**Published:** 2025-10-21

**Authors:** Alaa A. Fawzy, Marwa M. Raafat, R. Mahmoud, Omneya M. Helmy

**Affiliations:** 1 Department of Microbiology and Immunology, Faculty of Pharmacy, Future University in Egypt, Cairo, Egypt; 2 Department of Pharmacology, Toxicology and Supporting Science, College of Pharmacy- Al-Farahidi University, Baghdad, Iraq; 3 Microbiology and Immunology Department, Faculty of Pharmacy, Cairo University in Egypt, Cairo, Egypt

**Keywords:** Endophytes, Epiphytes, Quorum quenching, Lactonase, *Pseudomonas aeruginosa*, Biofilm, *Bacillus cereus*

## Abstract

**Background:**

*Pseudomonas aeruginosa* infections are often challenging to treat due to multiple drug resistance, besides the development of biofilms and a plethora of virulence factors regulated by quorum sensing. Quorum-quenching enzymes, such as N-acyl homoserine lactonases, represent a promising anti-virulence strategy by disrupting this signaling mechanism without exerting selective pressure, leading to resistance. This study aimed to screen endophyte and epiphyte isolates for lactonase activity and evaluate their potential to inhibit virulence in *Pseudomonas aeruginosa*.

**Results:**

Fifty-two bacterial isolates (42 endophyte and 10 epiphyte) were isolated from ten plants. The *aiiA* gene encoding lactonase enzyme was detected in 11 endophytes and one epiphyte isolate, among which nine showed complete degradation (100%) of the quorum sensing signal molecule N-hexanoyl-L-homoserine lactone. The partially purified lactonase enzyme from the endophyte *Bacillus cereus* AL1 isolate exhibited significant anti-virulence activity, reducing biofilm formation, swarming motility, and pyocyanin production against *Pseudomonas aeruginosa* PAO1 and clinical *Pseudomonas aeruginosa* isolates. Sequence alignment of the *Bacillus cereus* AL1 lactonase protein revealed close similarity to the homologous lactonase from *Bacillus cereus*. The quorum quenching activity of the partially purified lactonase AL1 provided protection in a *Galleria mellonella* infection model.

**Conclusion:**

The study highlights the potential of *Bacillus cereus* AL1 lactonase as an effective anti-virulence agent against *Pseudomonas aeruginosa* without the pressure for resistance development.

**Supplementary Information:**

The online version contains supplementary material available at 10.1186/s12866-025-04396-4.

## Background

Antibiotics combat bacterial infections by targeting essential cellular processes, but this exerts evolutionary pressure that fosters antibiotic resistance [1]. Antimicrobial resistance (AMR) has been acknowledged by the World Health Organization (WHO) as a leading global health crisis, ranking it among the three most urgent and critical threats to public health worldwide [2]. With few antibiotics in the pipeline, researchers are increasingly turning to new treatment approaches [3]. The anti-virulence therapy aims to attenuate the pathogenicity of bacteria rather than killing them, potentially minimizing resistance by lowering selective pressure [4].

*Pseudomonas aeruginosa (P. aeruginosa)* is a widely encountered Gram-negative bacterium known for its high incidence, characterized by its genetic diversity and remarkable capacity to develop multidrug resistance (MDR) [5]. It is involved in the onset of hospital-related infections such as urinary tract infections, pneumonia, and skin and soft tissue infections [6]. The WHO announced an updated list of MDR bacterial strains in 2024, in which *P. aeruginosa* and *Staphylococcus aureus* were classified as high-priority pathogens [7].

Quorum sensing (QS) constitutes a cell-to-cell signaling system mediated by population density that coordinates microbial community activities through small, diffusible autoinducer molecules (AIs) [8]. QS regulates multiple physiological functions, including plasmid transfer, virulence gene expression, biofilm formation, bioluminescence, production of exoenzymes, surface motility, and antibiotic resistance [9, 10]. In Gram-negative bacteria, the predominant QS mechanism is the LuxI/LuxR system, which operates through N-acyl L-homoserine lactone (AHL) signaling molecules. In this system, LuxI protein homologs synthesize AHLs, and when reaching a threshold concentration, they activate their cognate LuxR-family transcriptional regulators through direct binding to activate QS-controlled genes [11].

*P. aeruginosa* employs an intricate QS system centered around two key genetic elements: the lasRI and rhlRI operons. These encode LuxR-type transcriptional regulators that exhibit specific ligand binding. LasR recognizes N-(3-oxododecanoyl)-L-homoserine lactone (3-oxo-C12-HSL), while RhlR interacts with N-butyryl-L-homoserine lactone (C4-HSL) [12]. These systems collectively control the expression of genes responsible for biofilm formation, pyocyanin production, and swarming motility [13].

Since QS is non-essential for bacterial growth, disrupting it through quorum quenching (QQ) strategies can attenuate bacterial virulence without exerting bactericidal effects [14]. Inhibition of QS can be achieved by QS inhibitors or QQ enzymes. QQ enzymes degrading the AHLs are categorized into three primary types: lactonases, acylases, and oxidoreductases. Among lactones, the major identified families include phosphotriesterase-like lactonases (PLLs), metallo-β-lactamase-like lactonases (MLLs), and paraoxonases. These enzymes have a unified catalytic mechanism, although variations exist in the AHL substrate. Lactonase specificity is primarily determined by its active site and how well the substrate’s acyl chain fits within it [15]. Broad substrate specificity is common among most of the MLL lactonases identified so far: GcL, MomL, AidC, and aiiA [16]. AHL-lactonase, a member of the metallohydrolase superfamily, facilitates the hydrolytic breakdown of AHL signaling molecules by targeting and opening their lactone ring structure [17]. AHL lactonase AiiA24B1 is the earliest and most thoroughly studied enzyme [18]. Subsequently, homologs of *aiiA* lactonase have been identified in various bacteria and are prevalent in several *Bacillus* spp [19, 20]. QS in *P. aeruginosa* can be effectively disrupted by lactonases, resulting in a notable reduction in virulence, by inhibiting the production of essential virulence factors, such as pyocyanin and proteases, and disrupting biofilm formation [21, 22].

Endophytes are symbiotic microbes that colonize plant tissues without inducing disease, thereby contributing significantly to plant health and defense mechanisms. They enhance plant growth, improve stress resilience, and protect against pathogens [23]. Endophytes are abundant and found in a wide variety of plants, producing numerous bioactive compounds and enzymes such as lactonases that are valuable for biotechnological applications [24, 25]. Epiphytes are vital to the ecosystem as they support biodiversity, serve as habitats for diverse organisms, and aid in nutrient cycling [26]. A study by Ma et al. (2013) found that QQ bacteria were widely detected on tobacco leaves, exhibiting significant strain-level diversity, which highlights the considerable role of epiphytes in disrupting QS and protecting plants [27]. Previous studies have predominantly focused on QQ bacteria isolated from soil or rhizospheric environments [18], with limited attention to plant-associated endophytes and epiphytes as potential reservoirs of QQ enzymes [28]. The isolation of endophytic microorganisms from medicinal plants is a promising field, as they produce enzymes and bioactive metabolites, including secondary compounds with antidiabetic and anti-hypercholesterolemic effects [29]. Only a few studies have explored their bacterial populations, especially those with lactonase activity. Moreover, some investigations have either identified *aiiA* homologs or assessed crude enzyme preparations separately [30]. Few have integrated molecular screening with functional assays to validate lactonase-mediated anti-virulence effects [22].

In the current research, we aimed to screen endophytic and epiphytic bacteria isolated from various plants for the presence of the *aiiA* homologs and evaluate the efficacy of the partially purified lactonase (PP-Lactonase) enzyme in suppressing virulence in *P. aeruginosa* isolates. Our study is therefore distinct in exploring plant-associated bacteria from diverse medicinal and ornamental hosts, combining *aiiA* gene detection with the functional evaluation of PP-Lactonase against both standard and clinical MDR *P. aeruginosa* isolates, thereby addressing a critical gap linking genetic identification to clinically relevant anti-virulence efficacy.

## Methods

### Collection of plant samples

A total of ten plants were collected from the Orman Botanical Garden (30°01′45.12″ N, 31°12′47.16″ E) and the Research Institute of Plant Protection (30.046356° N, 31.207320° E), Giza, Egypt, in February 2023, and were included in this study. They comprised three ornamental and seven medicinal plants (Table 1). The collection was conducted with prior permission from the relevant authorities at both institutions and in accordance with national regulations governing plant material collection. Plant species were identified by expert botanists at the Research Institute of Plant Protection. Specimens of plants parts, including leaves, roots, and stems, were collected and transferred into sterile bags for laboratory analysis. All the plant species used in this study were not endangered or protected species.

**Table 1:** Endophytic and epiphytic bacterial isolates from the tested ornamental and medicinal plants

Description of Plants						Endophytic bacteria			Epiphytic bacteria		
No.	Name	Family	Plant code	Type of plant	Source	Isolate from leaves	Isolate from stems	Isolate from roots	Isolate from leaves	Isolate from stems	Isolate from roots
1	*Vitex purpurea*	Lamiaceae	PL-1	Ornamental	Orman Garden	B5, B3	B12, B2	0	Be1, Be3	0	Be4
2	*Vitex trifolia*	Lamiaceae	PL-2	Ornamental	Orman Garden	B1	0	0	0	0	0
3	*Ocimum tenuiflorum*	Lamiaceae	PL-3	Medicinal	Orman Garden	B7, B15	B12, B13, B14, B25	0	0	0	0
4	*Laurus nobilis*	Lauraceae	PL-4	Medicinal	Orman Garden	B23	B27	0	0	0	0
5	*Clerodendron splendens*	Lamiaceae	PL-5	Ornamental	Orman Garden	B6, B19	B21, B33	0	0	0	0
6	*Rosmarinus officinalis*	Lamiaceae	PL-6	Medicinal	Research Institute of Plant Protection	B16	B14, B20	B42	0	0	0
7	*Thymus vulgaris*	Lamiaceae	PL-7	Medicinal	Research Institute of Plant Protection	B17	B4, B8	B10, B18, B22, B39	0	0	0
8	*Origanum majorana*	Lamiaceae	PL-8	Medicinal	Research Institute of Plant Protection	B24, B26	B28, B29	0	0	0	0
9	*Ocimum basalicum*	Lamiaceae	PL-9	Medicinal	Research Institute of Plant Protection	B31	B35	B9, B32, B40	0	0	0
10	*Lavandula dentata*	Lamiaceae	PL-10	Medicinal	Research Institute of Plant Protection	B34, B37	B11, B41	B36	Be2, Be5, Be6, Be7	0	Be8, Be9, Be10

### Isolation of endophytic bacteria

The plant’s leaves, stems, and roots were carefully rinsed with running water to remove dirt and then allowed to air dry. The plant parts were surface sterilized [31]. Briefly, the samples were immersed in 70% (v/v) ethanol for 2 min, subjected to multiple rinses with sterilized water, dipped in a 0.25% sodium hypochlorite solution for 1 min, and subsequently rinsed again with sterilized water for 3–5 times. The samples were then aseptically cut into small segments using a sterilized sharp blade and placed onto Luria-Bertani (LB, Biomark, India) agar plates. The plates were incubated at 30 °C for 48 h to promote the growth of endophytic bacteria. Controls included culturing the last washing water to ensure the inhibition of epiphytes, while negative control included non-inoculated LB plates to ensure the sterility of the media. Single colonies were isolated and inoculated onto fresh LB agar media. Each culture was checked to confirm its purity, and the confirmed pure endophytes were maintained in LB broth with 20% glycerol at − 80 °C [32].

### Isolation of epiphytic bacteria

The healthy roots, leaves, and stems were washed and soaked in sterile 0.9% NaCl maintained at 37 °C under shaking conditions for 1 h. Serial dilutions with a tenfold dilution ranged from 10^⁻1^ to 10 ^−7^ in sterile 0.9% NaCl. Then, 0.1 ml from each dilution was inoculated on LB agar plates, and the plates were incubated at 30 °C for 2 days [33]. The purified epiphytes were stored in LB broth with 20% glycerol at − 80 °C [32].

### PCR screening for AHL-lactonase gene

DNA extraction from each bacterial isolate was performed using the QIAamp DNA Mini Kit (Qiagen, Germany), according to the manufacturer’s instructions. The presence of the lactonase gene (*aiiA*) in all bacterial isolates was screened through PCR, using the PCR master mix 2x FastTeq Premix (ToloBio, China) and *aiiA* primer pairs: *aiiA*-F2 (5′-CGGAATTCATGACAGTAAAGAAGCTTTA-3′) and *aiiA*-R2 (5′-CGCTCGAGTATATATTCAGGGAACACTT-3′) [20]. Lactonase-producing *Bacillus weihenstephanensis* P65 isolate was used as a positive control [34]. The cycling conditions were as follows: initial denaturation at 94 °C for 5 min, 5 cycles of 94 °C (45 s), 44 °C (45 s), 72 °C (1 min); 30 cycles of 94 °C (45 s), 53 °C (45 s), 72 °C (1 min), followed by final extension at 72 °C for 8 min [35]. The resulting PCR amplicons were examined by electrophoresis on 1.2% agarose gels [36].

### Preparation of bacterial cell-free supernatants

The bacterial cell-free supernatants (CFSs) were prepared from *aiiA*-positive bacterial isolates according to Ayyappan et al. (2022) with modifications. In summary, a single colony was introduced into a 250 mL Erlenmeyer flask containing 50 mL LB broth and incubated at 37 °C with shaking at 180 rpm for 24 h. The culture was then adjusted to an optical density of 600 nm (OD₆₀₀ = 1.0) and used as a 2% (v/v) inoculum in 250 mL double-strength LB broth in a 1 L Erlenmeyer flask, followed by incubation at 37 °C with shaking at 200 rpm for 48 h. The centrifugation of cultures was performed at 10,000 rpm for 30 min at 4 °C, and supernatants were filter-sterilized through 0.22 μm filters before storage at −20 °C [37]. The Bradford assay was employed to determine protein concentration, with bovine serum albumin used for standardization [38].

### Screening for a quorum quenching enzymatic activity using the agar well diffusion assay

The AHL inactivation assay was performed using the agar well diffusion technique with *Agrobacterium tumefaciens (A. tumefaciens)* KYC55 as reporter strain. The assay was conducted according to the method of Raafat et al. (2019) with some modifications [30]. Briefly, 1 mL of an overnight culture of *A. tumefaciens* KYC55 was inoculated into 50 mL of minimal medium supplemented with 100 µg/mL Gentamycin (Sigma- Aldrich), 4 µg/mL Tetracycline (Sigma- Aldrich), and 100 µg/mL Spectinomycin (Cayman, USA) and incubated overnight at 28–30 °C with shaking at 160 rpm. The prepared culture was mixed with 50 mL molten LB agar containing 1.2% agar, at 46 °C to prevent killing the used bacteria, and supplemented with the same antibiotics and 60 µg/mL X-Gal (Titan biotech ltd.) [39]. Twenty milliliters of the prepared agar culture were transferred into sterile petri dishes, and after solidification, wells of 10 mm diameter were bored into the medium. Fifty microliters of CFSs from *aiiA* positive isolates were incubated for 3 h with 50 µM N-hexanoyl-L-homoserine lactone (C6-HSL; Cayman, USA). After incubation, the enzymatic activity was terminated by heating the mixture at 95 °C for 10 min. Then, 50 µL of the reaction mixture was added per well. The positive control consisted of 50 µL of 50 µM C6-HSL, while 50 µL phosphate-buffered saline (PBS) served as the negative control. The plates were maintained at 28–30 °C for 12–18 h, after which the wells were examined for blue zones. A persistent blue zone confirms intact C6-HSL, whereas reduction or clearing around wells demonstrates AHL degradation.

### Confirmation of a lactonase degradation activity by the acidification test

Fifty microliters of CFSs from the isolates showing a clear or reduced blue zone around the wells in the agar well diffusion assay were incubated for 3 h with C6-HSL (50 µM). Then, 100 µL of 0.2 M HCl was added to acidify the reaction, and it was incubated overnight at 30 °C [27]. Fifty microliters of the acidified reaction were added to each well of the prepared agar culture. Fifty microliters of 50 µM C6-HSL and PBS were used as positive and negative controls, respectively, as previously described in the agar well diffusion assay. The plates were incubated at 28–30 °C for 12–18 h. The appearance of a blue zone indicates that the activity is due to a lactonase rather than an acylase enzyme, as the hydrolysis of the HSL ring by AHL-lactonase is a reversible process.

### Determination of the minimum inhibitory concentration of the prepared cell free supernatants against *P. aeruginosa*

The minimum inhibitory concentration (MIC) of crude (CFS) from the *aiiA* positive isolates against *P. aeruginosa* PAO1 and 10 MDR *P. aeruginosa* clinical isolates (P1-P10) was determined by the broth microdilution method [40]. All the MDR *P. aeruginosa* clinical isolates were from the culture collection of the Department of Microbiology and Immunology, Faculty of Pharmacy, Cairo University. Twofold serial dilutions of CFSs, starting at a concentration of 4 mg/mL, were prepared in LB medium. Subsequently, each well of a 96-well plate received 10 µL of the adjusted culture to achieve a final inoculum of approximately 5 × 10⁵ CFU/mL. Then the plates were incubated at 37 °C for 18 ± 2 h. Both positive and negative controls were included to ensure assay validity. The MIC value was defined as the lowest concentration of CFS that effectively suppressed visible bacterial growth. Triplicate measurements were obtained for all experimental conditions to confirm reproducibility.

### Screening the anti-virulence activity of the CFS of *aiiA*-positive isolates against *P. aeruginosa* PAO1

#### Anti-biofilm activity

The anti-biofilm effectiveness of CFS against *P. aeruginosa* PAO1 was evaluated using a static microtiter plate assay, following the methods described by Cady et al. (2012), with minor modifications [41]. *P. aeruginosa* PAO1 was grown in LB broth for 18 h at 37 °C with shaking at 180 rpm. The overnight culture was adjusted to approximately 1.5 × 10^7^ CFU/mL in LB medium. Then, 100 µL of the adjusted culture was mixed with 100 µL of the tested CFS (at a sub-inhibitory concentration) in a 96-well microtiter plate. Positive and negative controls were included, and the plate was kept at 37 °C for 18 h without agitation. The planktonic cells were transferred to a new microtiter plate, and the OD₆₀₀ was measured using a microtiter plate reader (ELISA microplate reader; BioTek, USA). Wells were washed three times with 150 µL of PBS. The formed biofilms were detected by staining with 100 µL of 0.1% (w/v) crystal violet (Alpha Chemika, India), followed by incubation for 15 min at room temperature and washing four times with PBS. Then 100 µL of ethanol (95%) was added to solubilize the crystal violet, transferred into a new microtiter plate, and the absorbance was measured at 595 nm. The assay was performed in triplicate. The ratio of OD 595/OD 600 was used to normalize the amount of biofilm formed to the growth of bacteria in the presence and absence of the enzyme. Then, the percentage of biofilm formation inhibition was calculated as follows [42]:

$$\begin{aligned}\mathrm{Biofilm}\;\mathrm{formation}\;\mathrm{inhibition}\;\%&=\mathrm{OD}595\;\mathrm{of}\;\mathrm{control}\;\\&-\mathrm{OD}595\;\mathrm{of}\;\mathrm{treated}/\mathrm{OD}595\;\mathrm{of}\;\mathrm{control}\;\\&\times100\end{aligned}$$

#### Swarming motility inhibition assay

Briefly, a 5 mL culture of *P. aeruginosa* PAO1 in Tryptic Soy Broth (TSB; Oxoid, UK) was incubated overnight at 37 °C with shaking at 180 rpm [43]. The culture was adjusted to 0.5 McFarland. Plates for swarming were prepared as follows: TSB media containing 0.5% agar were supplemented with 1 mg/mL of the crude enzyme (sub-inhibitory concentration). Then, 15–20 mL was poured into each petri dish [44]. Dried swarming agar plates were inoculated at the center with 10 µL of the adjusted *P. aeruginosa* PAO1 culture and incubated for 48 h [45]. Control plates without lactonase enzymes were included. The swarming behavior was assessed by observing the plates and measuring the diameter of the motility zone. The test was performed in triplicate. The percentage inhibition of motility was calculated as follows [42]:

$$\begin{aligned}\mathrm{Motility}\;\mathrm{Inhibition}\;\%=&\left[\left(\mathrm{motility}\;\mathrm{area}\;\mathrm{of}\;\mathrm{control}\;\right.\right.\\& \left.\left.-\mathrm{motility}\;\mathrm{area}\;\mathrm{of}\;\mathrm{treated}\right)\right.\\& \left. /\mathrm{motility}\;\mathrm{area}\;\mathrm{of}\;\mathrm{control}\right]\times100\end{aligned}$$

#### Pyocyanin production inhibition assay

Briefly, 5 mL of the overnight *P. aeruginosa* PAO1 cultures adjusted to a 0.5 McFarland were treated with 1 mg/mL of crude enzyme (CFS). A control culture without enzyme was included. All bacterial cultures were maintained at 37 °C for 48 h. After incubation, the bacterial culture was centrifuged at 8,000 rpm for 10 min to pellet the cells. The supernatant containing pyocyanin was subsequently extracted with 3 mL of chloroform, followed by back extraction into 1 mL of 0.2 M HCl, resulting in a pinkish-red solution. The test was repeated in triplicate. The amount of pyocyanin was measured spectrophotometrically by recording the OD at 520 nm [10]. The percentage inhibition of pyocyanin production was calculated using the formula:

$$\begin{aligned}&\mathrm{Inhibition}\;\mathrm{of}\;\mathrm{pyocyanin}\;\mathrm{production}\;\\&\left(\%\right)=\left[\left(\mathrm{AC}-\mathrm{At}\right)/\mathrm{AC}\right]\times100\end{aligned}$$, where AC is the OD of the control culture and At denotes the OD of the treated culture [42].

#### Preparation of PP-Lactonase enzyme

Partial enzyme purification was performed using ammonium sulfate precipitation, as described by Rajesh et al. (2015), with slight modifications [46]. In summary, the protein content in 1000 mL of CFS was precipitated using solid ammonium sulfate at concentrations ranging from 50% to 80% with stirring overnight at 4 °C. The resulting precipitate was collected by centrifugation at 18,000 rpm for 30 min at 4 °C. The 0.05 M Tris-HCl buffer (pH 7.5) was utilized to dissolve the pellets, followed by dialysis in a dialysis tube with a molecular weight cut-off of 13,000 Da against 250 mL of the same buffer, and stirring overnight at 4 °C. The solution was filtered through a 0.22 μm cellulose acetate syringe filter, and the protein content was determined using the Bradford reagent and adjusted to 1 mg/mL. The agar well diffusion assay was performed to confirm activity, as mentioned before. To select the optimum ammonium sulfate concentration for partial purification of lactonase, Sodium Dodecyl Sulfate Polyacrylamide Gel Electrophoresis (SDS-PAGE) was performed using a 12.5% polyacrylamide gel following the method of Laemmli in 1970 [47]. A standard molecular weight protein marker (Genetix Biotech Asia Pvt. Ltd., New Delhi, India) was used.

#### Determination of the minimum inhibitory concentration of PP-Lactonases and assessment of the anti-virulence activity againstPseudomonas aeruginosa

The MIC of the PP-Lactonases from selected *aiiA* positive isolates was determined against *P. aeruginosa* PAO1 and the 10 MDR *P. aeruginosa* clinical isolates using the broth microdilution method, as described earlier [40]. All the tested clinical isolates produced pyocyanin, showed swarming motility, and were moderate to strong biofilm producers.

PP-Lactonase was evaluated for its ability to inhibit biofilm formation, swarming motility, and pyocyanin production in *P. aeruginosa* PAO1 and the tested clinical isolates at a sub-inhibitory concentration, following the same methodology previously described for the crude enzyme.

Furthermore, the alteration of *P. aeruginosa* PAO1 biofilm structure by the CFS and PP-lactonase of the selected isolate, showing promising anti-virulence activity was examined using scanning electron microscopy (SEM) (Zeiss EVO 15, Germany). Biofilms of *P. aeruginosa* PAO1 and *P. aeruginosa* PAO1 treated with either CFS or PP-Lactonase were grown in 8-well chambered cover slides for 24 h, gently rinsed with phosphate buffer, and fixed with glutaraldehyde. The fixed samples were dehydrated using ethanol, sputter-coated with gold, and observed using SEM [48].

#### Molecular identification of the most promising anti-virulence Lactonase-producing isolate and its aiiA gene

The bacterial isolate with the highest anti-virulent activity was identified through sequencing of its 16S rRNA gene. Genomic DNA was extracted by the QIAamp DNA Mini Kit (Qiagen, Germany) according to the manufacturer’s instructions. PCR was performed using the PCR master mix 2x FastTeq Premix (ToloBio, China) and the universal primers 27 F: (5′-AGA GTT TGA TCC TGG CTC AG-3′) and 1492R (5′-ACG GCT ACC TTG TTA CGC TT-3 [35]. The PCR conditions included an initial denaturation at 94 °C for 4 min, followed by 35 cycles of 94 °C for 45 s, 54 °C for 45 s, and 72 °C for 1 min, and a final extension of 72 °C for 8 min. The *aiiA* gene of the promising isolate was also amplified by PCR using *aiiA* primers as previously mentioned.

The PCR products of the amplified 16S rRNA and *aiiA* genes were purified using a QIAquick PCR purification kit (Qiagen, Germany). The purified PCR products were sequenced by the Sanger’s method using a 3500 Genetic Analyzer at Clinilab, Cairo, Egypt.

The 16S rRNA DNA sequence was analyzed using NCBI’s BLASTn tool to identify closely related sequences, and the identified isolate’s sequence was deposited in GenBank. Sequences with high similarity were retrieved from the NCBI database and used for phylogenetic analysis. The *aiiA* gene sequence was translated using the Expert Protein Analysis System (ExPASy) tool, and the resulting lactonase amino acid sequence was also deposited in GenBank. Multiple sequence alignments of the lactonase with homologous AHL lactonases from the NCBI database were performed. Phylogenetic trees were generated using the neighbor-joining method with 1000 bootstrap replicates in MEGA version 11.0 [49].

##### Identification of the PP-Lactonase enzyme using liquid chromatography-mass spectrometry

The protein sample was prepared and digested according to standard procedures [50]. The peptide band was sent to the Children Cancer Hospital Foundation 57357 Basic Research Department, Proteomics and Metabolomics research program, Cairo, Egypt, for liquid chromatography-mass spectrometry **(**LC-MS) analysis [50]. Briefly, the SDS-PAGE band corresponding to ~ 28 kDa was excised, destained, and treated with a fixation solution comprised of 50% methanol and 12% acetic acid. Subsequently, gel fragments underwent three 15-minute washes with 200 µL of 50 mM ammonium bicarbonate (ABC) and 50% acetonitrile (ACN) on a shaker. The pieces were vacuum-dried until they appeared shrunk and white. For reduction, 10 mM dithiothreitol (DTT) was added, and the samples were incubated at 60 °C for 30 min. Alkylation was carried out using 55 mM iodoacetamide (IAA) in the dark for 30 min. The pieces were then washed, dehydrated with ACN, and vacuum-dried.

For trypsin digestion, 50 µL of trypsin (10 ng/µL) was added to the gel and incubated overnight at 37 °C with shaking at 600 rpm. Peptides were extracted by adding 80 µL of an ACN: MilliQ water: formic acid (66:33:1) solution, shaking for 5 min twice. The peptide extracts were collected, and the solvent was evaporated using a SpeedVac. The final peptides were reconstituted in 25 µL of 0.2% formic acid (FA). LC-MS analysis was conducted on an Eksigent nanoLC 400 autosampler interfaced with an Ekspert nanoLC 425 pump and a Sciex TripleTOF 5600 + mass spectrometer (SCIEX, Canada). A 10 µL peptide sample was injected, and the peptides were trapped on a CHROMXP C18CL 5 μm (10 × 0.5 mm) (SCIEX, Canada) cartridge at a flow rate of 10 µL/min using mobile phase A (LC-MS water containing 0.1% FA) for 3 min. The peptides were eluted using a gradient of mobile phase B (acetonitrile containing 0.1% formic acid) from 3% to 80% over 45 min at a flow rate of 5 µL/min. The analytical column was a 3 μm ChromXP C18CL, 120 Å, 150 × 0.3 mm column (SCIEX, Canada). Mass spectrometry was operated in positive ion mode, with high-resolution time-of-flight (TOF) MS scans followed by product ion scans of the 40 most intense precursor ions in a cycle time of 1.5 s. The TOF mass range was set to 400–1250 m/z, and the MS2 product ion range was from 170 to 1500 m/z. The ion selection threshold was 150 counts per second (cps), and the source voltage was maintained at 5500 V, with a curtain gas pressure of 10 psi. Calibration was performed using the SCIEX, Canada tuning solution (part number 4457953), and samples were randomly distributed within each batch for consistent analysis. The total analysis time was 55 min [50].

Data acquisition was conducted using Analyst TF 1.7.1 software (SCIEX, Canada). The raw MS data files; obtained from the TripleTOF™ 5600 + mass spectrometer; were processed using ProteinPilot (version 5.0.1.0, 4895) with the Paragon Algorithm (version 5.0.1.0, 4874). Protein identification was carried out by searching against two databases: the *B. cereus* database (Swiss-Prot and TrEMBL, containing 223,040 proteins) and a custom database for the “Quorum-quenching N-acyl-homoserine lactonase” protein (containing 96 accessions). The following search parameters were applied: cysteine alkylation was performed using iodoacetamide, and trypsin was used as the digestion enzyme. Gel-based identification was employed. Biological modifications were considered, and a false discovery rate (FDR) analysis was performed. Bias correction was applied to ensure accurate protein identification.

#### Evaluation of the anti-virulence activity of PP-Lactonase using Galleria mellonella infection model

The anti-virulence activity of PP-Lactonase of the isolate with high in vitro anti-virulence activity was evaluated against *P. aeruginosa* PAO1 using a *Galleria mellonella* larvae infection model [51]. Laboratory-reared larvae, weighing 150–250 mg, were obtained from the National Research Center, Giza, Egypt, and reared on artificial media [52].

The toxicity of the PP-Lactonase on *Galleria mellonella* larvae was tested [51]. Different enzyme concentrations (0.125, 0.0625, 0.03125, 0.0156, 0.0078 mg/mL) were prepared, and each of the five groups (*n* = 7 larvae per group) received 10 µL of the tested concentration injected into the hemocoel (below the hind proleg). Survival was monitored daily for 3 days, and the percentage mortality was recorded. The highest non-toxic concentration of the PP-Lactonase was used in the infection model. To determine the lethal infective dose of *P. aeruginosa* PAO1, 10 µL of inoculum ranging from 10^1^ to 10^9^CFU/mL was injected into the hemocoel (below the hind proleg) in each group of tested larvae (*n* = 7 larvae per group) [53]. For the infection model assay, a total of 21 larvae were divided into three groups (*n* = 7 per group): infected-treated, infected-untreated, and a negative control group. Systemic infection of *Galleria mellonella* larvae was carried out according to Peleg et al. (2009). A 10 µL of PAO1 inoculum and 10 µL of PP-Lactonase were pre-incubated together at 37 °C for 1 hour before injection into the infected-treated group. The infected-untreated group received 10 µL of PAO1 inoculum mixed with 10 µL sterile saline. For the negative control group, 20 µL of sterile saline was injected. After injection, the larvae were incubated aerobically at 28 ± 2 °C, and the survival was monitored daily for 72 h. Larvae showing no reaction upon touch were classified as dead. The experiment was performed in triplicate.

### Statistical analysis

Data is expressed as the mean of three independent replicates ± standard deviation (SD). The standard error and statistical analysis were performed using GraphPad Prism 10.1 (La Jolla, CA, USA) software to determine statistical significance; differences between means were assessed using an unpaired t-test and one-way ANOVA, followed by Tukey’s post-hoc multiple comparison test. Statistical significance was defined as a *p-*value < 0.05. For the *Galleria mellonella* survival assay, Kaplan–Meier survival curves were generated, and statistical differences between groups were assessed using the Gehan–Breslow–Wilcoxon test.

## Results

### Isolation of bacterial endophytes and epiphytes

A total of ten plants, comprising three ornamental and seven medicinal, were identified by a qualified expert. Fifty-two bacterial isolates were recovered. A total of 42 endophytes were isolated from stems (*n* = 18), leaves (*n* = 15), and roots (*n* = 9). In addition, 10 epiphytes were obtained from leaves (*n* = 6) and roots (*n* = 4) (Table 1).

### Screening of lactonase-encoding gene by PCR

**Fig. 1:**
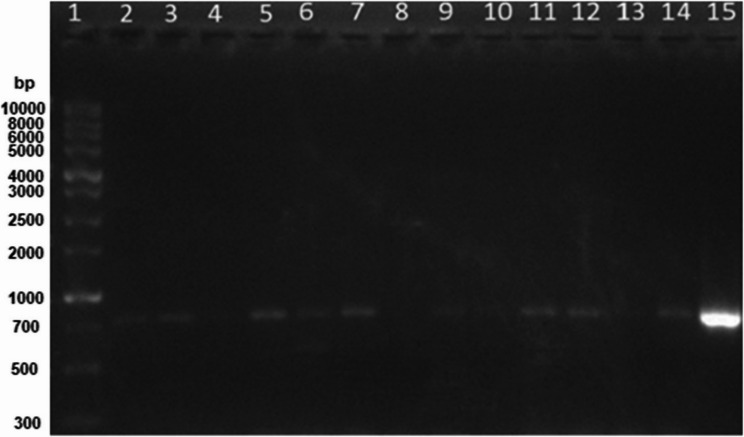
Agarose gel showing the PCR amplification products of the *aiiA* in the tested isolates. Lane 1 shows 1Kb DNA ladder (BioDyne, Tartu, Estonia); lanes 2, 3, 5, 6, 7, 9, 11, 12, and 14 show positive *aiiA* with the expected size of 750 bp; lanes: 4, 8, and 10 show the absence of *aiiA*; lane 15 show positive *aiiA* of the positive control (*Bacillus weihenstephanensis)* and lane 13 shows the negative control with no band

Using PCR, the bacterial isolates were screened for the presence of the AHL-lactonase (*aiiA*) gene. The expected amplicon size (~ 750 bp) was detected in twelve isolates (*n* = 12/52) (Fig. 1). The *aiiA* positive isolates comprised eleven endophytes and one epiphyte.

### Screening and confirmation of lactonase activity

**Fig. 2:**
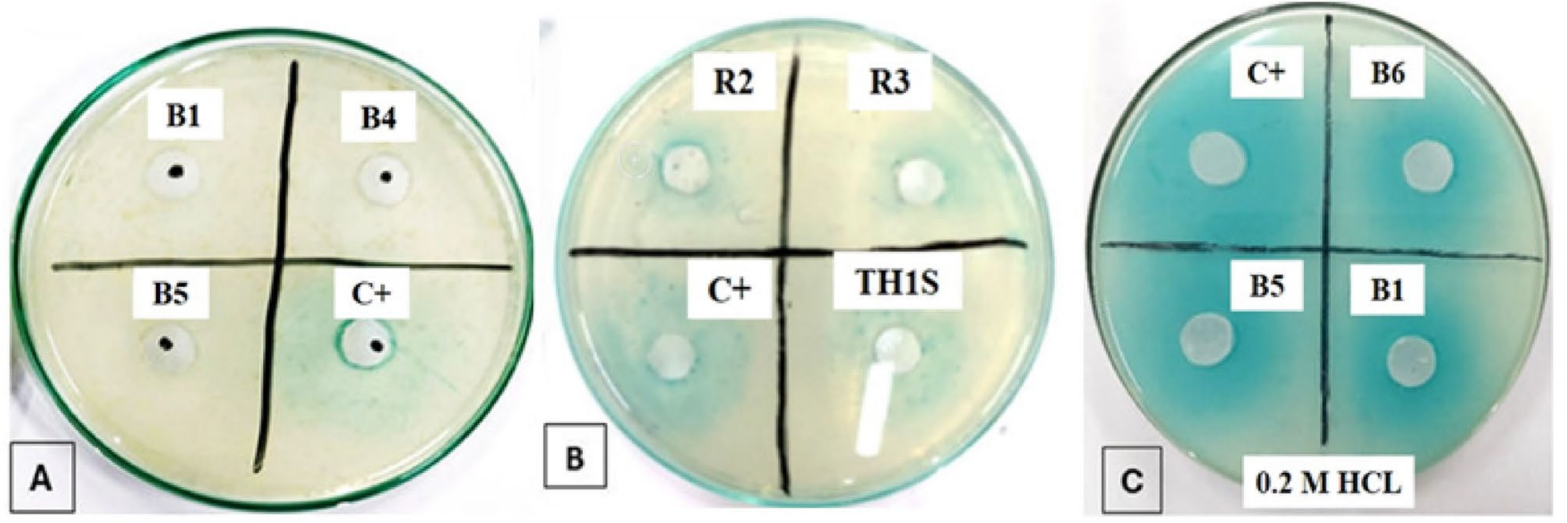
Evaluation of C6-HSL signal degradation by bacterial CFS using agar well diffusion assay. (**A)** Complete degradation of the AHL signal by CFSs of B1, B4, and B5 with the absence of blue zone; (**B**) Partial degradation of the AHL signal by CFSs of R2, R3 and TH1S with blue zones smaller than the control, and (**C**) Restoration of blue zones of B1, B5 and B6 after incubation with 0.2 M HCl confirm the presence of the lactonase enzyme. C + is the positive control not containing CFS and displaying a distinct blue zone

The ability of CFSs from the 12 *aiiA*-positive isolates to degrade C6-HSL was assessed using an agar well diffusion assay with *A. tumefaciens* KYC55 serving as a biosensor. A total of (*n* = 9/12) CFSs showed complete degradation of the tested signal (Fig. 2A). The remaining CFSs (*n* = 3/12) showed partial degradation of the signal (Fig. 2B). Furthermore, all 12 CFSs restored the blue zones around the wells after incubation with the tested AHL signal under acidic conditions using 0.2 M HCl, confirming a lactonase mediated QQ activity (Fig. 2C).

### The anti-virulence activity of the tested CFSs against *P. aeruginosa* PAO1

The nine bacterial CFSs that demonstrated complete degradation of the C6-HSL signal were selected to evaluate their potential to inhibit key virulence factors of *P. aeruginosa* PAO1 at sub-MIC concentrations. The MIC of the tested CFSs against PAO1 and MDR isolates was determined to be 2 mg/mL. Based on this result, a sub-inhibitory concentration equivalent to 0.5× MIC (1 mg/mL) was selected for use in subsequent experiments. The targeted virulence factors included swarming motility, biofilm formation, and the production of pyocyanin pigment. Among the tested CFSs, only the CFSs of B4 and B9 inhibited all three tested virulence factors of *P. aeruginosa* PAO1, while the remaining CFSs inhibited only one or two factors (Table 2). Notably, B4 and B9 CFSs were the only ones capable of inhibiting *P. aeruginosa* PAO1 biofilms. Accordingly, these two isolates were selected for further experiments.

**Table 2:** The anti-virulence activity of the tested CFSs against *P. aeruginosa* PAO1

Code of CFS	Biofilm inhibition %	Swarming motility inhibition %	Pyocyanin inhibition %
B1	0 ± 0.0	35 ± 3.5	31.1 ± 3.11
B4	38 ± 3.8	25 ± 2.5	15 ± 1.5
B5	0 ± 0.0	0 ± 0.0	15.5 ± 1.55
B6	0 ± 0.0	0 ± 0.0	70.6 ± 7.06
B8	0 ± 0.0	20 ± 2	20.4 ± 2.04
B9	48 ± 4.8	30 ± 3	19.6 ± 1.96
B10	0 ± 0.0	30 ± 3	85.5 ± 8.55
B11	0 ± 0.0	25 ± 2.5	42.4 ± 4.24
Be2	0 ± 0.0	0 ± 0.0	7.8 ± 0.78

^*^All experiments were performed in triplicate, and values are expressed as mean ± SD

### Partial purification of lactonase enzyme and detection of its molecular weight using SDS-PAGE

**Fig. 3:**
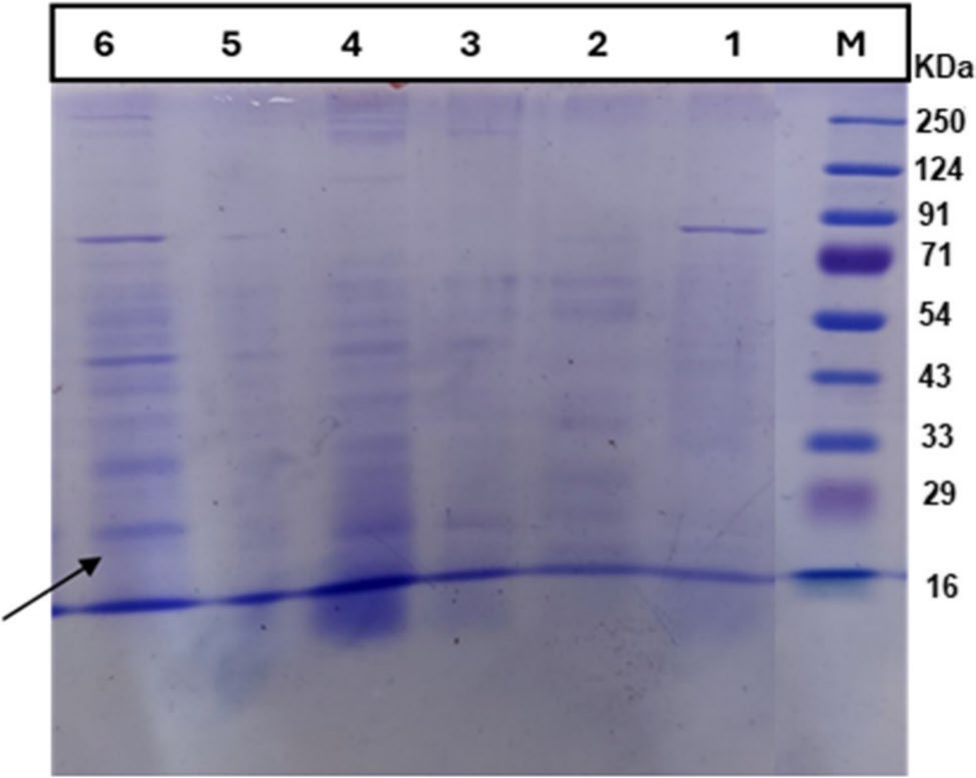
Detection of lactonase enzyme using different ammonium sulfate saturation by SDS-PAGE. Lane M shows the protein marker; lane 1 shows the crude extract of B4; Lanes 2, 3, 4, 6 show the PP-Lactonase yielded by precipitation using 50%, 80%, 70% and 60% ammonium sulfate saturation, respectively. The arrow in lane 6 marks the sharpest band corresponding to the expected size of the lactonase enzyme at about 28 kDa

**Fig. 4:**
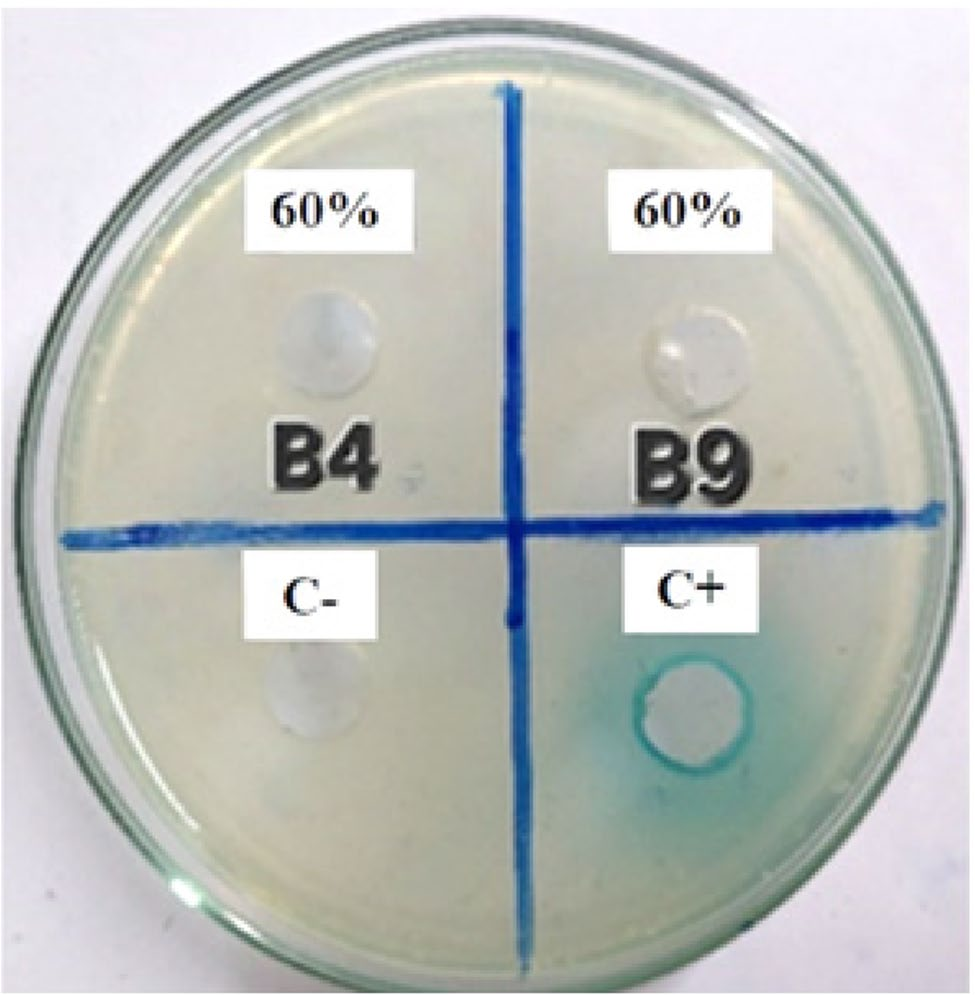
Detection of lactonase activity of B4 and B9 CFSs after partial purification using 60% ammonium sulfate saturation. Zones of activity were observed in B4 and B9 PP-Lactonase, indicating complete degradation of AHLs. The negative control (C-) showed no activity, while the positive control (C+) confirmed the assay validity. Clear zones suggest lactonase-mediated QQ activity

The two selected CFSs of B4 and B9 were subjected to partial purification using ammonium sulfate precipitation. The impact of ammonium sulfate saturation was assessed using concentrations ranging from 50% to 80%. The 60% ammonium sulfate saturation yielded the most distinct band on SDS-PAGE at the expected molecular weight of approximately 28 kDa (Fig. 3). Complete degradation of the C6-HSL signal in the agar well diffusion assay confirmed the presence of lactonase enzyme in the prepared PP-Lactonase enzyme (Fig. 4).

### The anti-virulence activity of B4 and B9 PP-Lactonase against P. aeruginosa PAO1

**Fig. 5:**
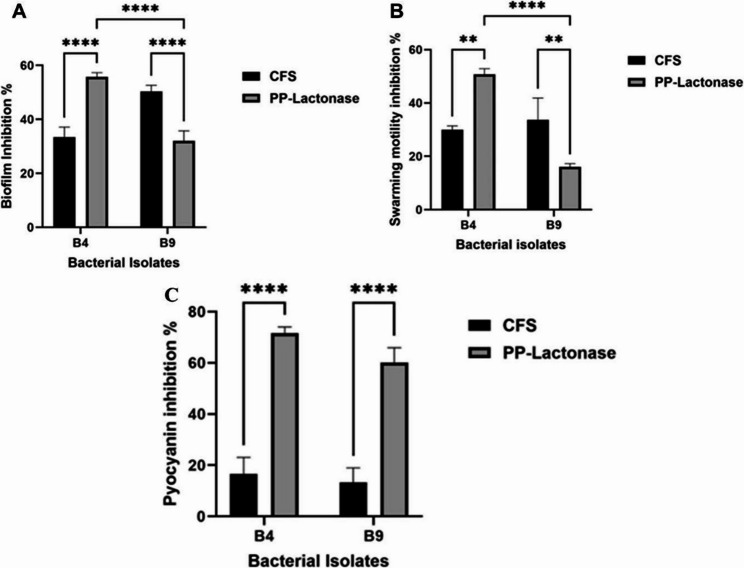
The effect of B4 and B9 CFSs and PP AHL-lactonases on virulence factors in *P. aeruginosa* PAO1. (**A)** Percentage of biofilm inhibition; (**B**) Percentage of swarming motility inhibition; (**C**) Percentage of pyocyanin production inhibition. Error bars represent standard deviations from three independent experiments. The unpaired t-test was used to determine significance; the asterisks refer to statistically significant differences as follows: (**) *p*-value < 0.01, and (****) *p*-value < 0.0001

A comparative analysis evaluated the anti-virulence activities of the CFSs and PP-Lactonases from B4 and B9 isolates against *P. aeruginosa* PAO1 at a sub-MIC concentration standardized to 1 mg/mL total protein. The B4 PP-Lactonase demonstrated significantly enhanced inhibition of biofilm formation compared to its CFS (*p*-value < 0.0001). In contrast, the B9 PP-Lactonase did not exhibit superior activity relative to its CFS (Fig. 5A). Similarly, the B4 PP-Lactonase showed a marked increase in swarming motility inhibition compared to its CFS (*p*-value < 0.01) (Fig. 5B; Fig. S1), while no notable difference was observed between the B9 PP-Lactonase and its CFS (Fig. 5B). Moreover, both B4 and B9 PP-Lactonases resulted in significantly greater inhibition of pyocyanin production than their respective CFSs (*p*-value < 0.0001) (Fig. 5C).

**Fig. 6:**
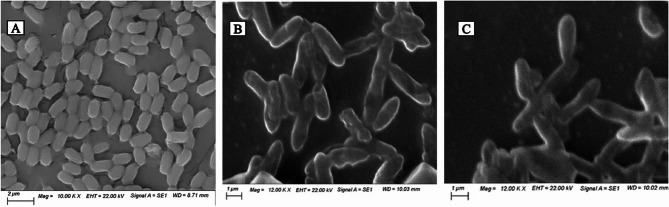
Scanning electron microscope images showing inhibition of biofilm formation by *P. aeruginosa* PAO1 treated with the cell free supernatant and PP-Lactonase of B4. (**A)** Untreated *P. aeruginosa* PAO1 control on glass coverslips captured at 10,000× magnification; (**B**) *P. aeruginosa* PAO1 treated with B4 CFS on glass coverslips captured at 12,000× magnification; (**C**) *P. aeruginosa* PAO1 treated with B4 PP-lactonase on coverslips captured at 12,000× magnification

The effect of both CFS and PP-Lactonase of B4 on the biofilm formation by *P. aeruginosa* PAO1 was further examined using SEM analysis. *P. aeruginosa* PAO1 produced a dense and continuous biofilm layer with intact bacterial cells (Fig. 6A). In contrast, *P. aeruginosa* PAO1 treated with either B4 CFS or PP-Lactonase produced biofilms that appeared markedly disrupted, with scattered bacterial cells and impaired structural integrity (Fig. 6B and C).

### Identification and phylogenetic analysis of the B4 bacterial isolate

**Fig. 7:**
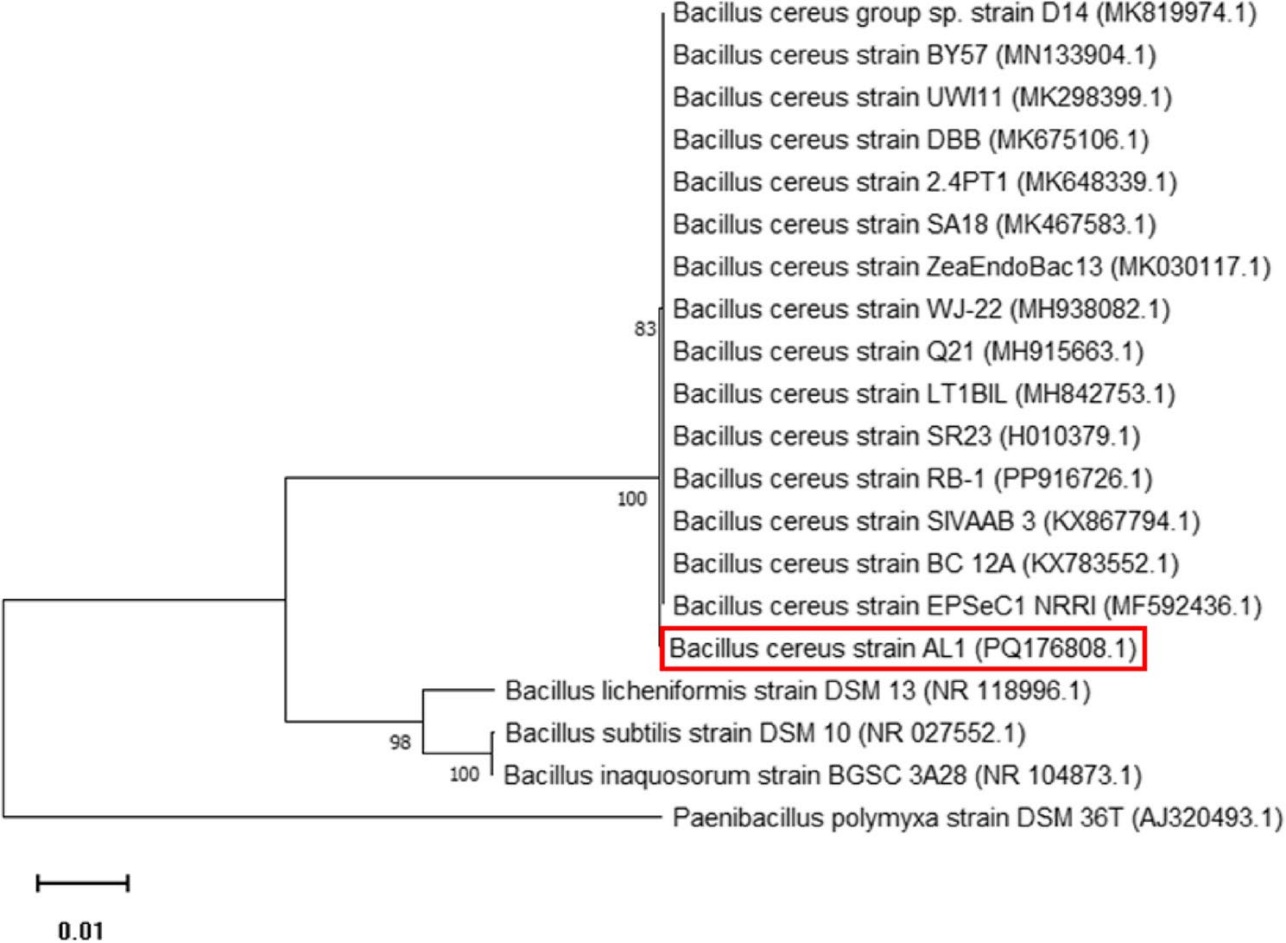
The phylogenetic tree of *B. cereus* strain AL1 (B4). This analysis was conducted using the MEGA software version 11, based on the 16S rRNA sequence and employing the Neighbor-joining method with 1000 bootstrap replicates

The B4 isolate, exhibiting the highest inhibitory activity against the key virulence factors of *P. aeuroginosa* PAO1, was identified as *B. cereus* strain AL1 through 16S rRNA gene amplification and sequencing. The amplified gene sequence was deposited in GenBank with accession number PQ176808 and is available at the following URL: https://www.ncbi.nlm.nih.gov/nuccore/PQ176808. BLAST analysis revealed 99.87% identity with *B. cereus* strain EPSeC1 NRRI (GenBank accession no. MF592436). To further establish the evolutionary relationship, a phylogenetic tree was generated using MEGA version 11, confirming the identification (Fig. 7).

### Phylogenetic analysis and sequencing of the lactonase from B. cereus strain AL1 (B4)

**Fig. 8:**
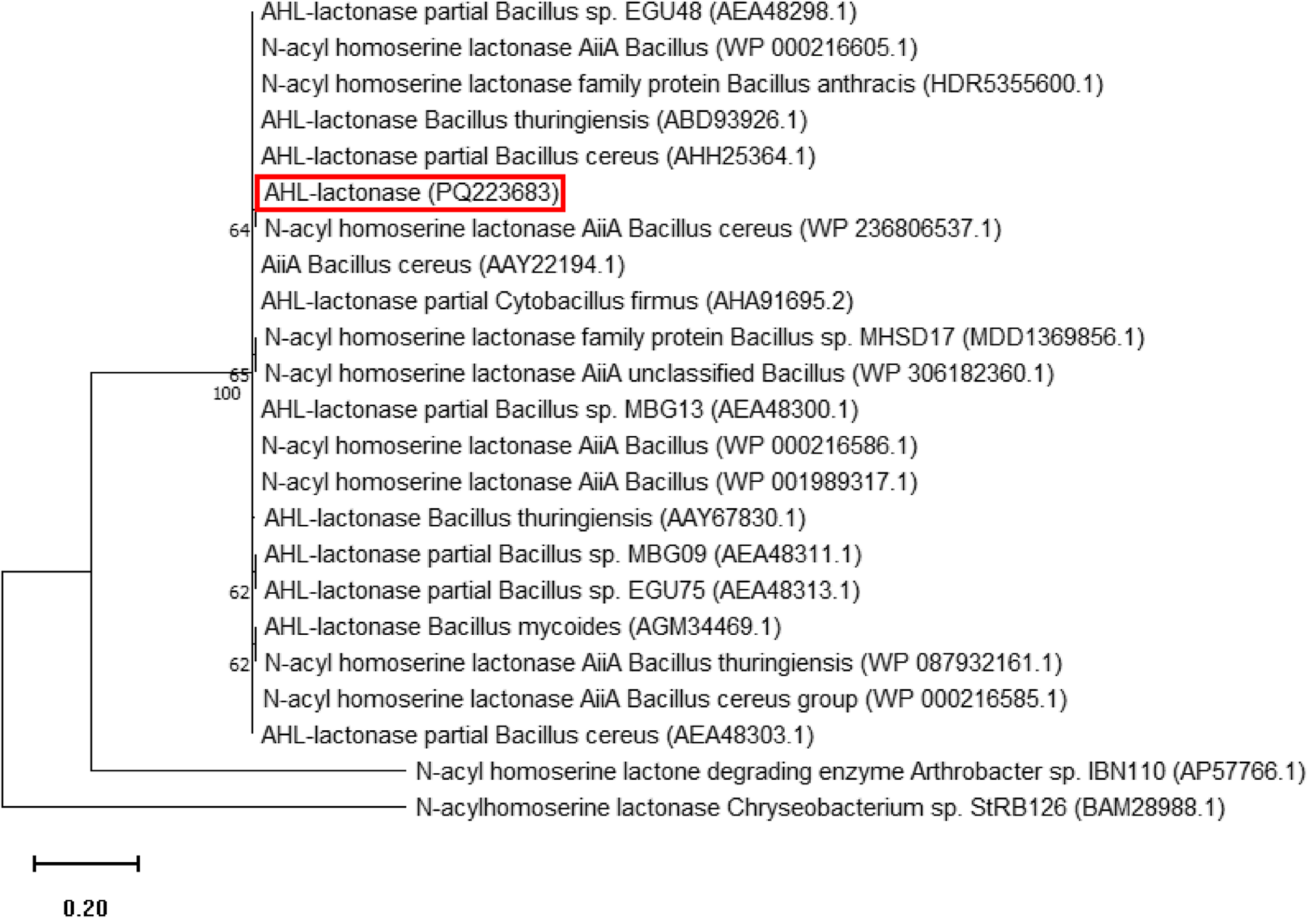
Phylogenetic tree of AHL-lactonase of *B. cereus* strain AL1 (B4). This was constructed using the MEGA package version 11, based on *B. cereus* strain AL1 (B4) lactonase protein sequence PQ223683 and the closest BLAST hits, using the Neighbor-joining method, with 1000 bootstrap replicates

The *aiiA* gene from the endophyte *B. cereus* strain AL1 (B4) was amplified by PCR and subsequently sequenced. The resulting nucleotide sequence was translated into an amino acid sequence and submitted to GenBank. The accession number PQ223683 has been assigned and is available at the following URL: https://www.ncbi.nlm.nih.gov/nuccore/PQ223683.1/. The translated amino acid sequence was aligned with known AHL-degrading lactonases from *Bacillus* spp., retrieved from the NCBI database. Phylogenetic analysis of the amino acid sequence indicated a close clustering with lactonases belonging to diverse *Bacillus* spp. (Fig. 8).

### Validating the anti-virulence activity of sub-MIC of *B. cereus* AL1 PP-Lactonase (B4) against MDR *P. aeruginosa* clinical isolates

**Fig. 9:**
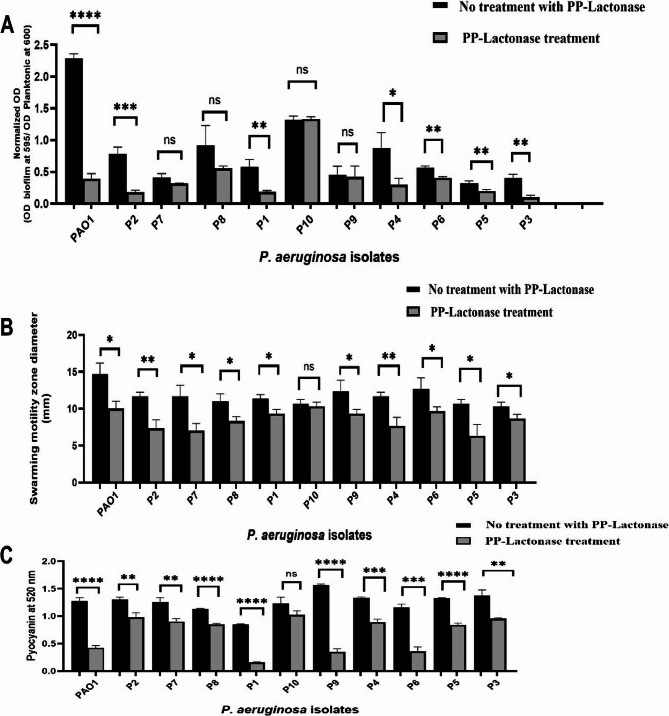
Effect of PP-Lactonase of *B. cereus* AL1 (B4) on virulence factors of the tested *P. aeruginosa* isolates. This panel illustrates the effects PP-Lactonase on each virulence factor in treated and untreated cultures of the tested *P. aeruginosa* isolates. (**A)** Biofilm formation data were normalized for accurate comparison. (**B)** The swarming motility zone diameter was measured in each isolate. (**C)** Pyocyanin production was detected by measuring the OD at 520 nm for each isolate. Error bars represent standard deviations from three independent experiments. Student’s *t*-test was used to determine statistical significance; the asterisks indicate statistically significant differences as follows: (*) *p*-value < 0.05, (**) *p*-value < 0.01, (***) *p*-value < 0.001, (****) *p*-value < 0.0001, and ns (non-significant)

The anti-virulence activity of PP-Lactonase of *B. cereus* AL1 (B4) was assessed against *P. aeruginosa* PAO1 and clinical biofilm-forming isolates. Biofilm inhibition was evaluated using the static microtiter plate assay. To eliminate any potential impact of the enzyme on bacterial growth, the OD of both planktonic cells and biofilms was measured and normalized for accurate comparison. Treatment with PP-Lactonase led to a marked decrease in biofilm formation in six clinical isolates, whereas four isolates showed a non-significant decrease (Fig. 9A). The percentage of biofilm inhibition ranged from 40.8% to 78.6%. Swarming motility inhibition was also evaluated in *P. aeruginosa* PAO1 and MDR clinical isolates. Significant inhibition was observed in ten isolates, while only one isolate showed a non-significant reduction (Fig. 9B). The percentage inhibition of swarming motility ranged from 29.3% to 65.1%. Additionally, the effect of AL1 PP-Lactonase (B4) on pyocyanin production was assessed by measuring absorbance at 520 nm. Pyocyanin production demonstrated a statistically significant decrease in ten isolates, while one isolate showed a non-significant change (Fig. 9C). The percentage of pyocyanin inhibition ranged from 24.3% to 81.5%.

### Detection of the quorum quenching N-acyl-homoserine lactonase protein of *B. cereus* AL1 (B4) using LC-MS

**Fig. 10:**
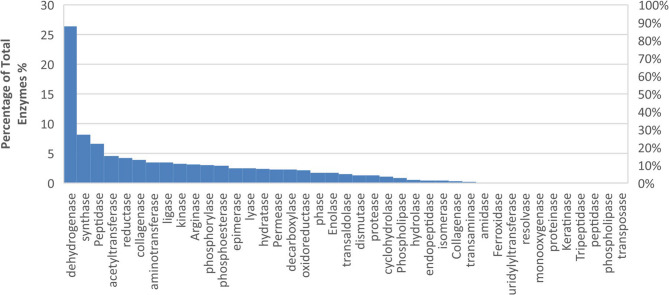
Functional categorization of enzymes identified in the LC-MS analysis of *B. cereus* proteome. A total of 91 proteins were identified from one gel band. The enzyme functions were classified based on their catalytic activities, and their relative abundances were calculated as a percentage of the total identified proteins

Using LC-MS analysis and ProteinPilot software, 91 proteins were identified from the excised gel band with a size of 28 kDa. These proteins were detected with high confidence (FDR < 1%) through a search against the *B. cereus* organism database (Swiss-Prot and TrEMBL). The identified proteins represented diverse functional categories (Fig. 10). The dominant enzyme classes identified include dehydrogenases, peptidases, synthases, acetyltransferases, kinases, and reductases, which play essential roles in metabolic processes such as oxidation-reduction reactions, peptide bond cleavage, biosynthetic pathways, phosphorylation, gene regulation, and electron transfer reactions. Additionally, two proteins were confirmed to correspond to the “QQ N-acyl-homoserine lactonase” enzyme. These proteins were analyzed against a custom database containing 96 accessions specific to this enzyme. Both proteins yielded significant matches, suggesting the presence of lactonase isoforms or variants. These variants belonged to the *B. cereus* spp. The identified proteins exhibited varying sequence coverages, ranging from 2.8% to 12.66%, based on unique peptide matches. Five distinct lactonase variants were identified, with their respective accession numbers, gene annotations, and percentage coverage. The protein with the highest sequence coverage (12.66%) corresponds to the entry A0A2H4Q762, annotated as a QQ N-acyl-homoserine lactonase fragment. Other variants, such as A0A2B3TZ42, A0A1S9TM12, A0A0G8F3P3, and A0A164P808, exhibited lower sequence coverage but were consistent with the functional identification of lactonase enzymes. Each variant was matched with at least one unique peptide, confirming the presence of these lactonase enzymes in the analyzed samples.

### Inhibition of *P. aeruginosa* PAO1 virulence in *Galleria* *mellonella* larvae infection model using the *B. cereus* AL1 (B4) PP-Lactonase

**Fig. 11:**
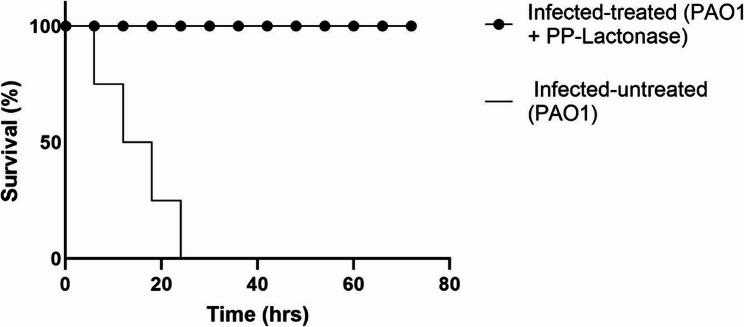
Protective effect of *B. cereus* AL1 (B4) PP-Lactonase on *Galleria mellonella* infected with *P. aeruginosa* PAO1. Three groups of *Galleria mellonella* larvae (7 larvae/group) were tested. Infected-treated group was injected with pre-incubated PAO1 inoculum and PP-Lactonase (0.125 mg/mL) of *B. cereus* AL1 (B4); while the infected-untreated group was injected with PAO1 inoculum mixed with sterile saline. In the negative control group, larvae were injected with sterile saline. The survival was monitored daily for 72 h

The QQ activity of *B. cereus* AL1(B4) PP-Lactonase was assessed in vivo through an infection model using *Galleria mellonella* larvae. To determine the appropriate infectious dose, varying concentrations of *P. aeruginosa* PAO1 were tested to evaluate dose-dependent mortality. An inoculum of 1.5 × 10⁷ CFU/mL was selected as it resulted in 100% mortality after 24 h. All tested doses of PP-Lactonase at sub-MIC levels were non-toxic to the larvae and did not affect larval viability. Therefore, the highest non-toxic dose was selected for subsequent in vivo experiments. The pre-incubation of PAO1 with AL1 (B4) PP-Lactonase at 0.125 mg/mL significantly attenuated bacterial virulence, resulting in 100% larval survival for 72 h (Fig. 11).

## Discussion

*P. aeruginosa* possesses numerous virulence factors that play key roles in its disease-causing capability. The search for anti-virulence agents targeting key pathogenic mechanisms in *P. aeruginosa* could be an alternative to traditional antibiotics [54]. Endophytes isolated from medicinal plants have shown significant potential as a source of necessary industrial and medical enzymes. For example, endophytic bacteria derived from *Kalanchoe daigremontiana* and *Cichorium intybus* have been identified as producing elevated levels of α-amylase and protease [55]. Similarly, endophytes derived from *Anredera cordifolia* demonstrated significant antioxidant and antibacterial activities specifically against *B. cereus* and *S. aureus* [56]. Medicinal plants such as *Artemisia afra*, *Aloe vera*, and *Tulbaghia violacea* have been shown to harbor QQ-active endophytes capable of inhibiting QS-regulated behaviors in pathogens like *P. aeruginosa* and *S. aureus* [57]. Moreover, plant-derived compounds such as hydroxytyrosol from *Terminalia phanerophlebia* exhibit synergistic QS inhibition effects, suggesting a co-evolutionary role between host plants and their microbiota in defense mechanisms [58]. These interactions not only enhance the medicinal value of the plants but also offer sustainable biocontrol strategies and novel sources of anti-infective agents. Our work aims to explore endophytes and epiphytes for lactonase QS inhibitors against *P. aeruginosa* and to evaluate their ability to suppress key virulence factors in *P. aeruginosa* clinical isolates. In our study, a total of 52 bacterial isolates were recovered, comprising 42 endophytic and 10 epiphytic bacteria. Epiphytic bacteria were less diverse, with only 10 isolates recovered from two plants. This limited distribution may be attributed to the specific microhabitats, seasonal variations, and surface characteristics of these plants. This can significantly influence the composition and prevalence of epiphytic microbial populations, affecting bacterial colonization and survival [59].

The AHL-lactonase encoded by *aiiA* degrades a wide range of AHL molecules, making it useful in QQ [60]. This broad-spectrum activity is advantageous for developing biocontrol agents that can target multiple QS-regulated mechanisms. In our study, 23% (*n* = 12/52) of the tested isolates were PCR-positive for *aiiA*, with 92% of these being endophytes. The primers used previously amplified *aiiA* from soil and marine water samples [30, 61]. The AHL-lactonase exhibits high activity across various AHL synthetic signals. According to catalytic efficiency, variations in the acyl chain length and substitutions do not significantly affect the lactonase’s performance. A study conducted using recombinant AHL-lactonase and its four variants, after purification and analysis of the kinetics and substrate specificity, revealed that AHL-lactonase had no or little residue activity to non-acyl lactones and non-cyclic esters, but displayed strong enzymatic activity toward all tested AHLs, varying in length and nature of the substitution at the C3 position of the acyl chain. AHL-lactonase displays the highest activity against C6-HSL, with C6-HSL being the preferred substrate among the reduced AHL molecules, while 3-oxo-C10-HSL is the best substrate among the C3-substituted AHL signals [17, 34]. The AHL-lactonase activity of CFSs from *aiiA-*positive isolates was evaluated for their capability to degrade C6-HSL utilizing the agar well diffusion assay, employing *A. tumefaciens* KYC55 as the biosensor [62]. The agar well diffusion assay, in combination with *A. tumefaciens* KYC55 biosensor, has been validated in several studies and is a widely accepted approach for assessing AHL-lactonase activity, and is considered a semi-quantitative method that offers a simple and reproducible technique for detecting AHL degradation [30, 63–65]. *A. tumafaciens* KYC55 exhibits high sensitivity toward diverse AHL derivatives, enabling broad-spectrum detection [66].

In our study, 75% (*n* = 9/12) of the CFSs of *aiiA*-positive isolates completely degraded the C6-HSL signal, and 25% of the tested CFSs showed varying degrees of degradation for the C6-HSL signal. These findings align with a prior study by Alramadhan. et al. (2023), who reported that 27% of *Bacillus* strains demonstrated the ability to fully degrade C6-HSL [67]. Another study reported that the potato root surface-associated strains of *Chryseobacterium* degrade various AHLs, including C6-HSL, with varying degrees of efficiency [68]. The restoration of blue zones around the wells after incubation of lactonase with the tested HSL signals under acidic conditions confirms the presence of lactonase enzymes and rules out acylase enzymes [69, 70]. This was observed in 100% of the CFSs, confirming lactonase activities.

Three essential virulence factors: biofilm formation, swarming motility, and pyocyanin production, were tested in this study, given their pivotal roles in *P. aeruginosa* pathogenicity and their modulation by QS. The CFSs of B4 and B9 exhibited inhibitory effects against all three virulence factors in *P. aeruginosa* PAO1, while the remaining CFSs inhibited one or two of the virulence factors. Similarly, a previous study has reported the inhibition of all three virulence factors by thermostable lactonases, such as SacPox, in *P. aeruginosa*, which hydrolyzes acyl-HSLs, key signaling molecules in QS systems [71].

Ammonium sulfate precipitation is a frequently utilized approach for protein purification in both large-scale and laboratory settings due to the high solubility, low cost, and ability to stabilize protein structures [72]. The impact of ammonium sulfate saturation on partial purification of B4 and B9 CFSs was assessed using different concentrations. The 60% ammonium sulfate saturation was optimal for precipitating the lactonase enzyme while minimizing the presence of other proteins, as evidenced by a more distinct band at the expected molecular weight of approximately 28 kDa in SDS-PAGE. This finding aligns with several studies [17, 73]. A comparative analysis of the anti-virulence activity of CFSs and PP-Lactonases of B4 and B9 was conducted on the three tested virulence factors of *P. aeruginosa* PAO1. B4 PP-Lactonase demonstrated a stronger inhibitory effect on biofilm formation than its CFS, suggesting that increased purity and concentration enhance its potency. However, the B9 CFS and PP-Lactonase exhibited biofilm inhibition, with PP-Lactonase achieving only 32% inhibition. Swarming motility in *P. aeruginosa* is a coordinated surface movement regulated by QS and facilitated by flagella and rhamnolipids. This behavior enhances surface colonization, biofilm formation, and virulence, contributing to antibiotic resistance and infection severity [74]. The B4 PP-Lactonase exhibited a superior inhibitory effect on swarming motility compared to its CFS. Pyocyanin is a blue-green pigment belonging to the phenazine class, produced by *P. aeruginosa* and regulated by the QS system. It acts as a virulence factor by inducing oxidative stress through the production of reactive oxygen species, leading to host tissue damage and immune suppression [75]. Anti-virulence agents possessing QS inhibitory activity, such as sesamol, have been reported to suppress *P. aeruginosa* biofilm formation markedly, downregulate the expression of QS- and virulence-associated genes, and alter antioxidant enzyme activity, thereby reducing virulence factor production and enhancing oxidative stress [10]. The B4 and B9 PP-Lactonase enzymes showed significant inhibitory effects on pyocyanin production by *P. aeruginosa* PAO1. Discrepancies in biofilm formation and swarming motility between B9 CFS and its PP-Lactonase indicate that additional factors or co-factors may influence their activity or that purification may have altered enzyme functionality, and this aligns with Sakr et al. (2018), who reported a higher inhibitory effect of crude Ahl-1 compared to its purified form [76]. These findings highlight the role of purification in determining enzyme efficacy.

The phylogenetic analysis of the amino acid sequence revealed that the lactonase from *B. cereus* AL1 (B4) clustered closely with lactonases from diverse *Bacillus* spp. Similarly, Nusrat et al. (2011) found that 45% of the tested *Bacillus* strains positive for *aiiA* were identified as *B. cereus* [20]. The lactonase gene has also been identified in various *Bacillus* spp [19, 77, 78]. LC-MS was utilized for protein identification and characterization in the SDS-PAGE gel band that exhibited the expected molecular weight, with a focus on QQ N-acyl-homoserine lactonase enzymes. This method enables the precise detection of proteins based on their mass-to-charge ratios. LC-MS analysis, combined with ProteinPilot software, confirmed that two proteins are lactonase isoforms or variants. These findings align with Bruhn et al. (2004), who demonstrated the effectiveness of the method in proteomic characterization and enzyme identification, such as QQ enzymes [79].

In our study, the PP-Lactonase of *B. cereus* AL1 (B4) exhibited significant anti-virulent activity against *P. aeruginosa* PAO1 and MDR *P. aeruginosa* clinical isolates. Regarding *P. aeruginosa* PAO1, PP-Lactonase of *B. cereus* AL1 achieved a 58.7% reduction in biofilm formation, which is consistent with previous reports of over 80% inhibition using various lactonases [70, 80]. On the contrary, Djokic et al. (2022) reported a lower percentage of biofilm formation inhibition (25%) using the YtnP-ZP1 lactonase at its highest tested concentration [81]. When tested against *P. aeruginosa* clinical isolates, the PP-Lactonase of *B. cereus* AL1 (B4) demonstrated a broad range of biofilm inhibition (40.8% to 78.6%). These results are consistent with previous studies that reported biofilm inhibition in *P. aeruginosa* isolates using crude lactonase preparations, with inhibition percentages ranging from 47% to 86% [30] and 19.7% to 55.7% [76]. Swarming motility in the tested *P. aeruginosa* isolates was also significantly inhibited by PP-Lactonase of *B. cereus* AL1 (B4), with percentage inhibition ranging from 29.3% to 65.1%. These findings support earlier reports on the impact of *aiiA* expression in *P. aeruginosa* PAO1 on motility inhibition [21, 82]. The inhibition of pyocyanin production by PP-Lactonase of *B. cereus* AL1 (B4) was evident in *P. aeruginosa* isolates, with percentage inhibition ranging from 24.3 to 81.5%. Human paraoxonase 1 (hPON1) significantly reduces pyocyanin levels [83], while *Bacillus*-derived lactonase achieved up to 70% inhibition in pyocyanin production [80]. Furthermore, recombinant Ahl-1 achieved percentage inhibitions ranging from 48 to 75.9% against MDR *P. aeruginosa* strains [76]. However, lactonase enzymes, such as YtnP-ZP1, were reported to reduce elastase activity without affecting pyocyanin production [81]. Collectively, these findings highlight the broad-spectrum potential of the PP-Lactonase of *B. cereus* AL1 (B4) as an effective QQ enzyme capable of attenuating multiple virulence determinants in *P. aeruginosa*, positioning it as a promising candidate for anti-virulence therapy. The *Galleria mellonella* larvae serve as a reliable in vivo model for studying bacterial infections due to their immune system’s similarity to vertebrates [84]. In our study, pre-treatment with the highest non-toxic dose of the PP-Lactonase *B. cereus* AL1 (B4) effectively attenuated *P. aeruginosa* PAO1 virulence and improved *Galleria mellonella* larval survival up to 72 h. These findings align with previous studies in which QQ enzymes such as lactonase (YtnP) and acylase (PvdQ) have been shown to reduce bacterial virulence and enhance larval survival with *Serratia marcescens* [59] and *Burkholderia* infections [85].

## Conclusion

This study highlights the significance of endophytic bacteria from medicinal plants as valuable sources of QQ enzymes. The PP-Lactonase of *B. cereus* AL1 (B4) significantly inhibited virulence in *P. aeruginosa* PAO1 and clinical *P. aeruginosa* isolates via inhibition of biofilm formation, pyocyanin production, and swarming motility. Pretreatment of *P. aeruginosa* PAO1 with the PP-Lactonase of *B. cereus* AL1 (B4) resulted in 100% survival in the *Galleria mellonella* larvae infection model. Further investigations should focus on the complete purification and cloning of the *B. cereus* AL1 (B4) lactonase to facilitate large-scale enzyme production, improve the stability of the purified enzyme, and assess its performance in complex infection models. Additionally, investigating the *B. cereus* AL1(B4) purified lactonase interaction with diverse AHL signals and evaluating its potential synergy with conventional antimicrobial agents could expand its applicability as an anti-virulence strategy against MDR *P. aeruginosa* infections and other pathogens.

## Supplementary Information

Supplementary Material 1.

Supplementary Material 2.

Supplementary Material 3.

## Data Availability

Data availability section was added and the direct links for the amplified gene sequence deposited in GenBank with accession number PQ176808 and the accession number PQ223683 are included in this published article.
