# Supplementary material for: Quorum quenching by endophytic Bacillus cereus AL1: a lactonase-based anti-virulence strategy against Pseudomonas aeruginosa
Source: BMC Microbiol. 2025 Oct 21;25:669. doi: 10.1186/s12866-025-04396-4 (PMC12539043; doi:10.1186/s12866-025-04396-4)
Supplement: Supplementary file 1 — Supplementary Material 1. [file 12866_2025_4396_MOESM1_ESM.docx]

**Supplementary file**

**Quorum Quenching by endophytic *Bacillus cereus* AL1: A Lactonase-Based Anti-Virulence Strategy against Pseudomonas aeruginosa**


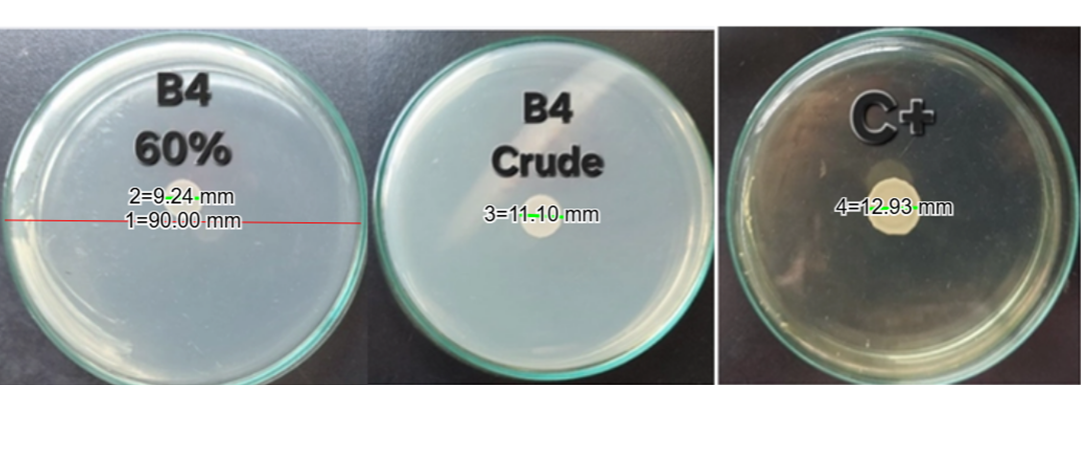


**Fig. S1** The effect of B4 CFS and PP-Lactonase on swarming motility. PP-Lactonase exhibited a significantly greater inhibitory effect than the crude extract when compared to the positive control (C+).
