# Supplementary material for: Quorum quenching by endophytic Bacillus cereus AL1: a lactonase-based anti-virulence strategy against Pseudomonas aeruginosa
Source: BMC Microbiol. 2025 Oct 21;25:669. doi: 10.1186/s12866-025-04396-4 (PMC12539043; doi:10.1186/s12866-025-04396-4)
Supplement: Supplementary file 3 — Supplementary Material 3. [file 12866_2025_4396_MOESM3_ESM.pdf]

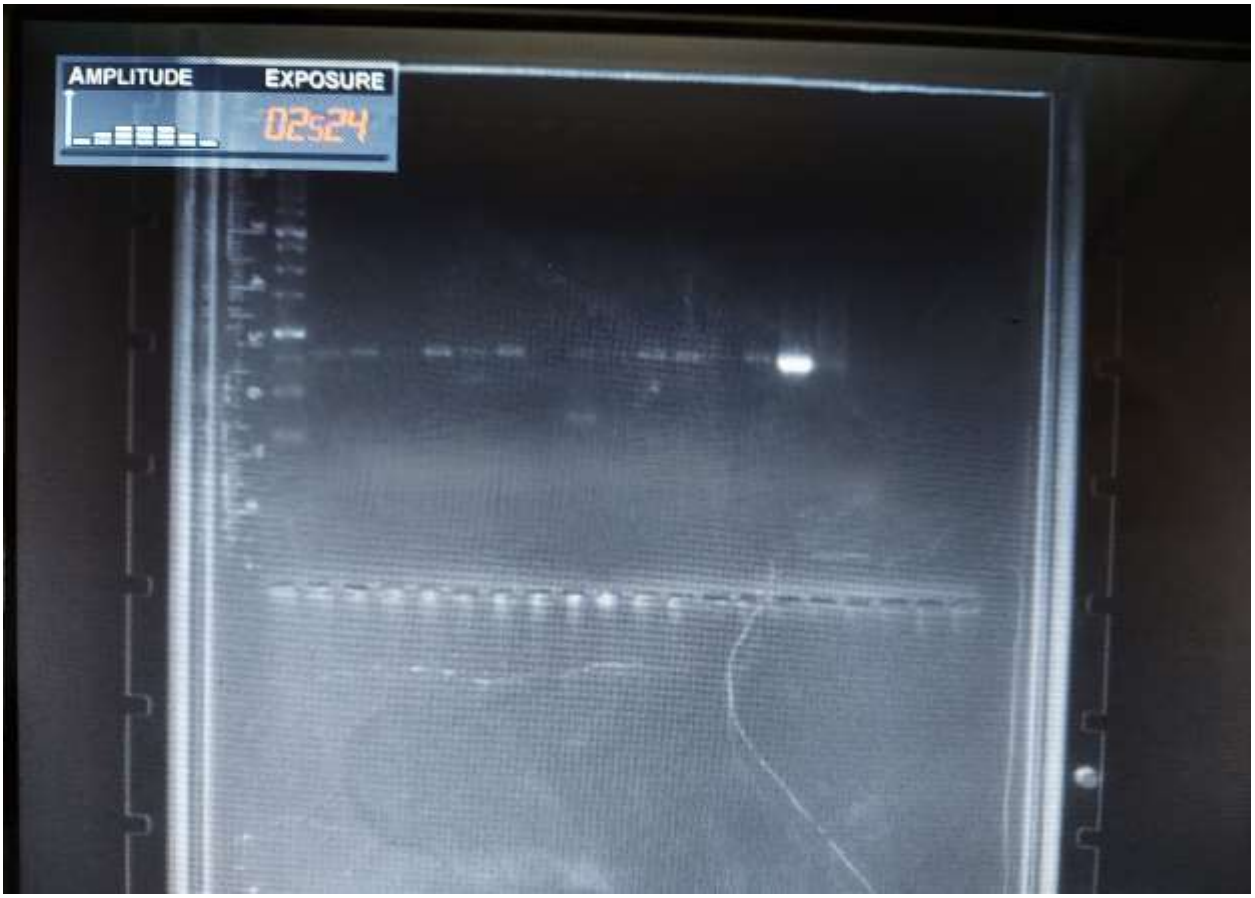

Fig. 1 Agarose gel showing the PCR amplification products of the *aiiA* in the tested isolates. Lane 1 shows 1Kb DNA ladder (BioDyne, Tartu, Estonia); lanes 2, 3, 5, 6, 7, 9, 11, 12, and 14 show positive *aiiA* with the expected size of 750 bp; lanes: 4, 8, and 10 show the absence of *aiiA*; lane 15 show positive *aiiA* with the expected size of 750 bp of the positive control (*Bacillus weihenstephanensis)* and lane 13 shows the negative control.

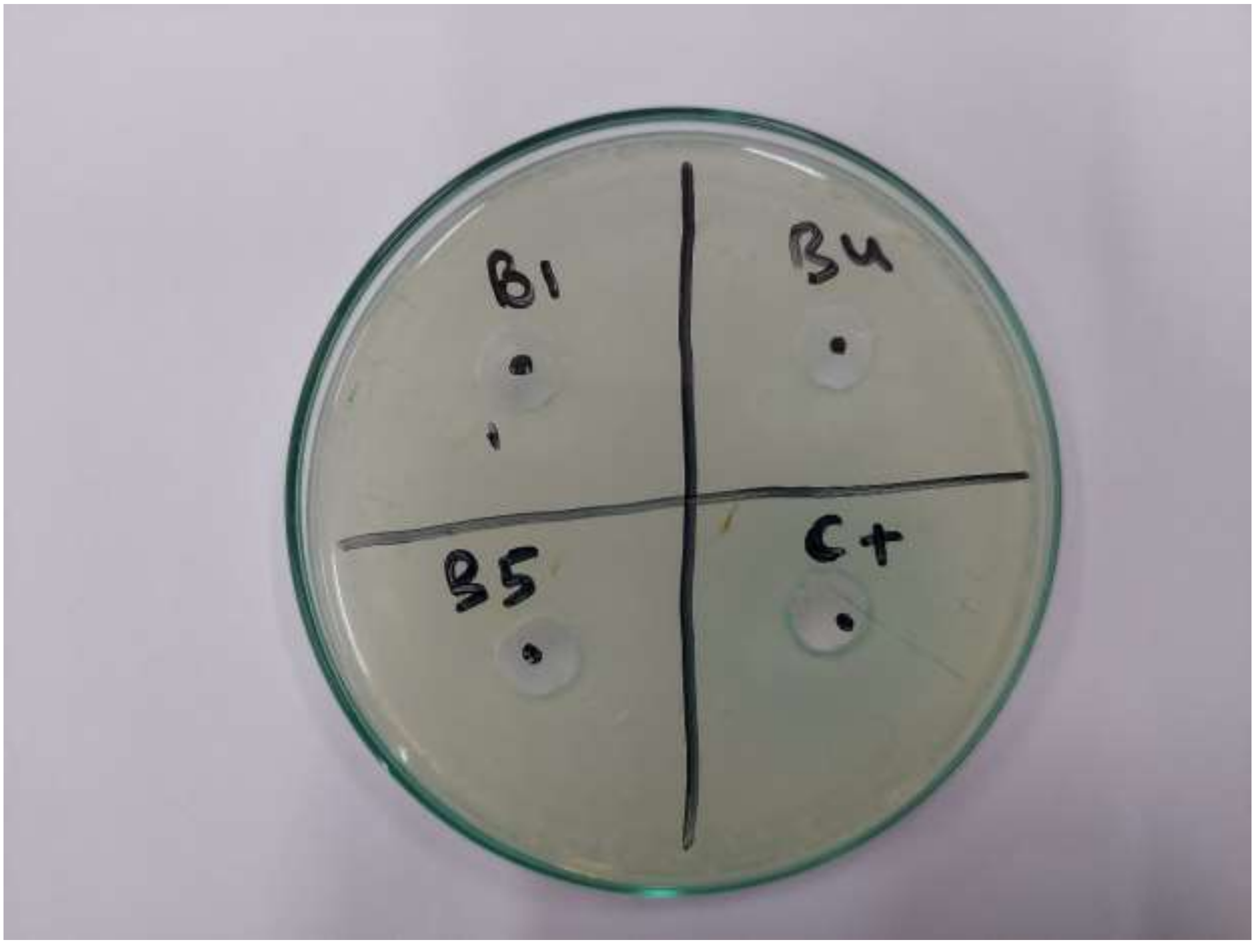
B1
B4
B5
C+

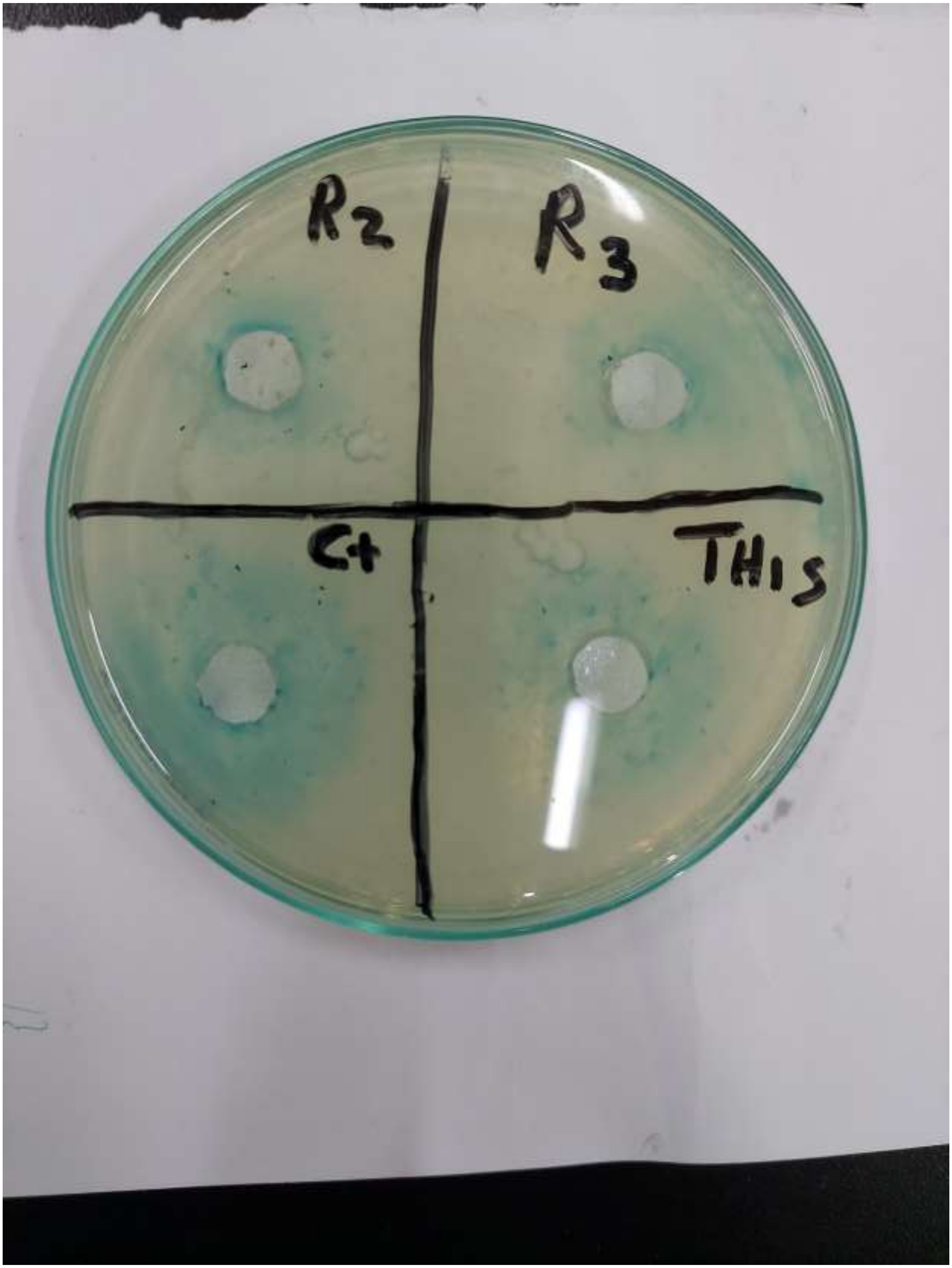
R2
R3
C+
THIS

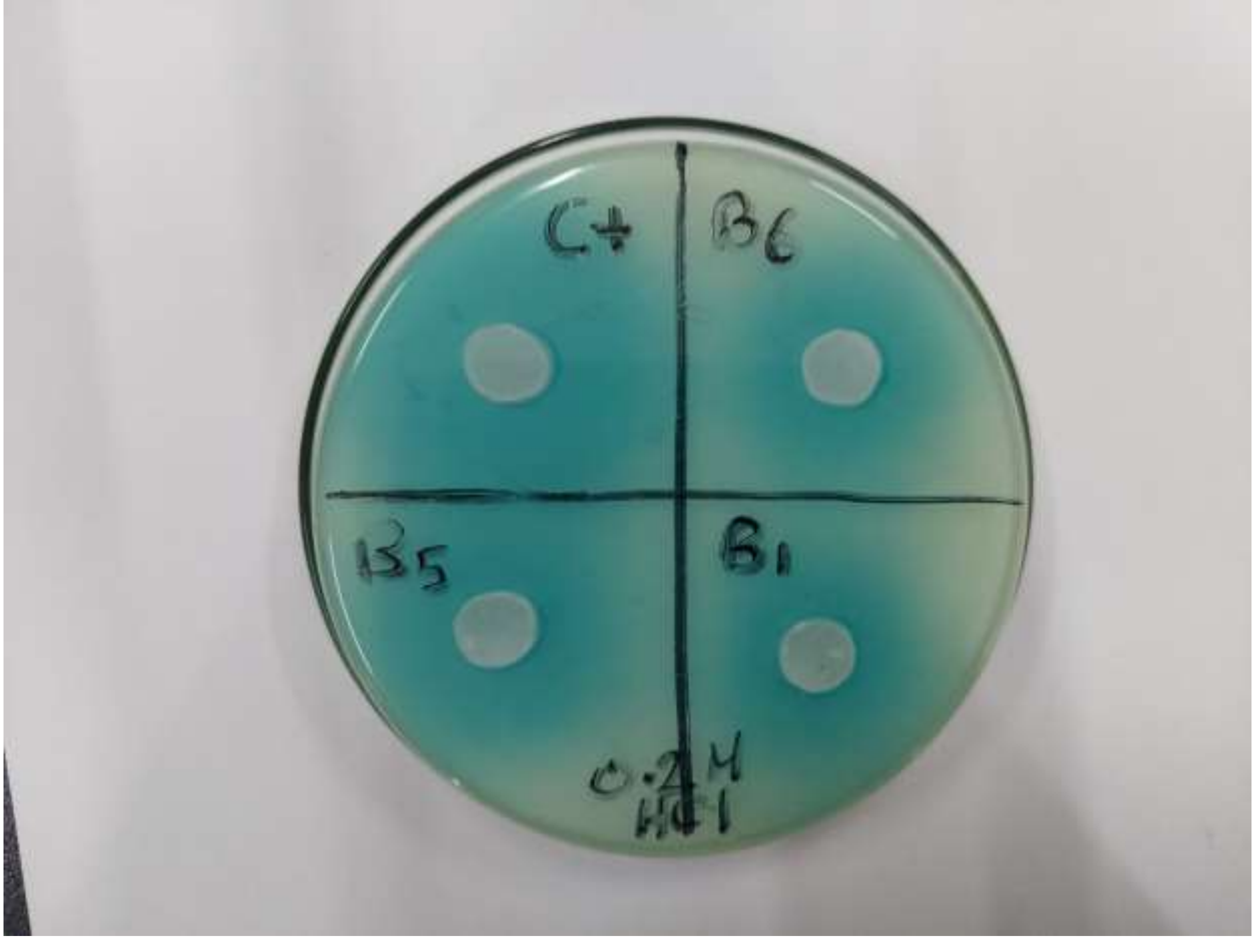

Fig. 2 Evaluation of C6-HSL signal degradation by bacterial CFS using agar well diffusion assay. (**A**) Complete degradation of the AHL signal by CFSs with the absence of blue zone; (**B**) partial degradation of the AHL signal by CFSs with blue zones smaller than the control, and (**C**) the restoration of blue zones after incubating with 0.2 M HCl indicates the presence of the lactonase enzyme. C+ (Positive control) not containing CFS, displaying a distinct blue zone.

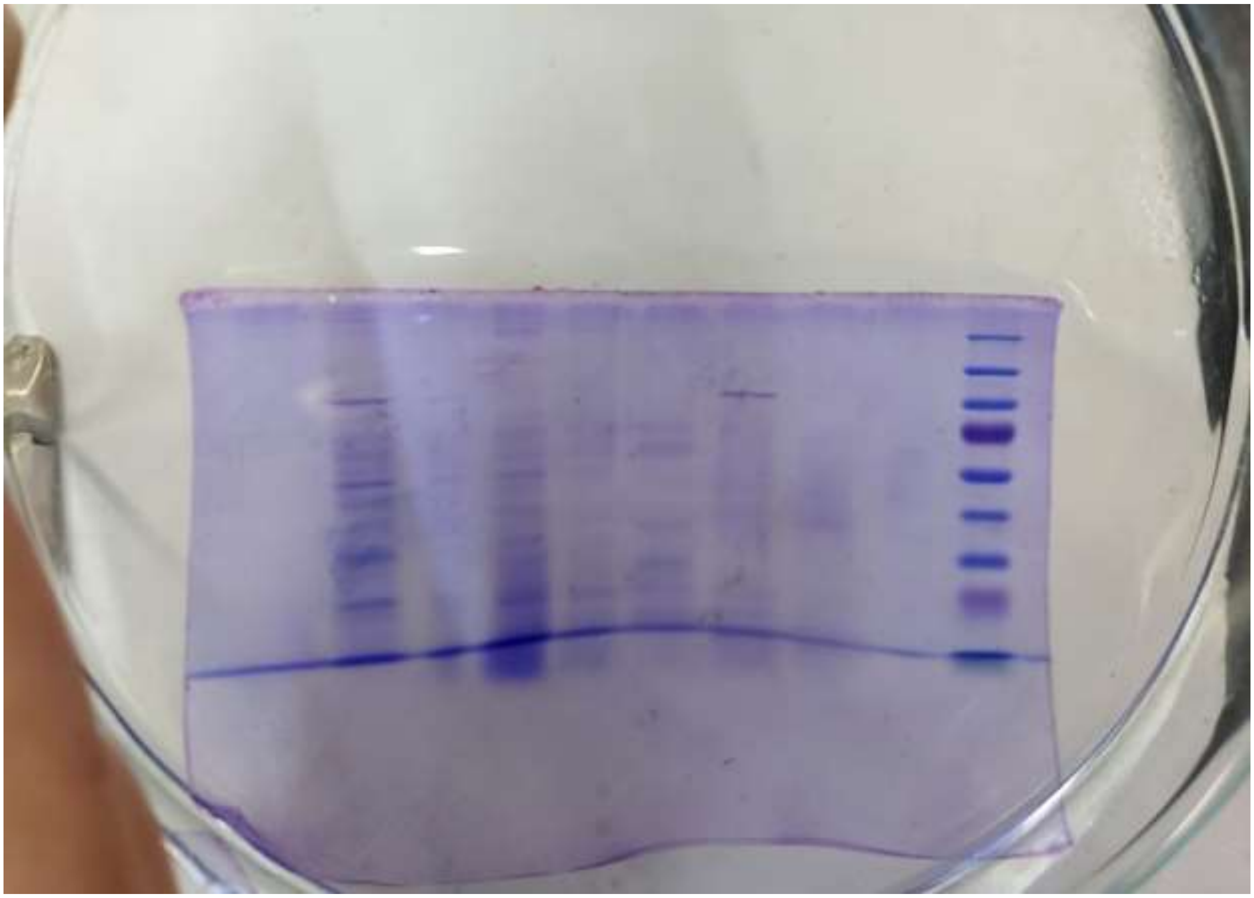

Fig. 3 Detection of lactonase enzyme using different ammonium sulfate saturation by SDS-PAGE. Lane M shows the protein marker; lane 1 shows the crude extract of B4; Lanes 2, 3, 4, 6 show the PP-Lactonase yielded by precipitation using 50%, 80%, 70% and 60% ammonium sulfate saturation, respectively. The arrow in lane 6 marks the sharpest band corresponding to the expected size of the lactonase enzyme at about 28 kDa.

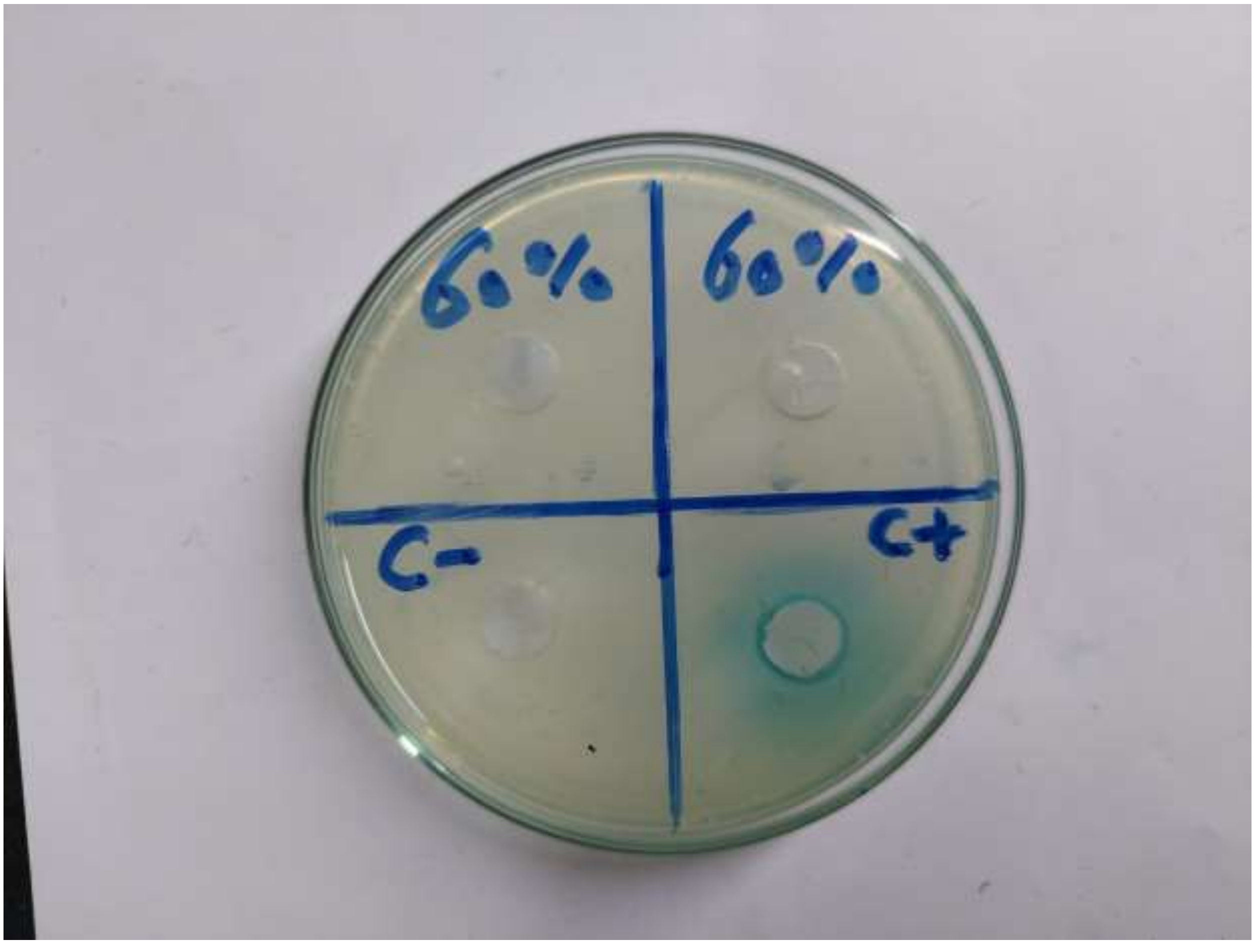

Fig. 4 Detection of lactonase activity after partial purification using 60% ammonium sulfate saturation. Zones of activity were observed in B4 and B9 PP-Lactonase, indicating complete degradation of AHLs. The negative control (C-) showed no activity, while the positive control (C+) confirmed the assay validity. Clear zones suggest lactonase-mediated QQ activity**.**

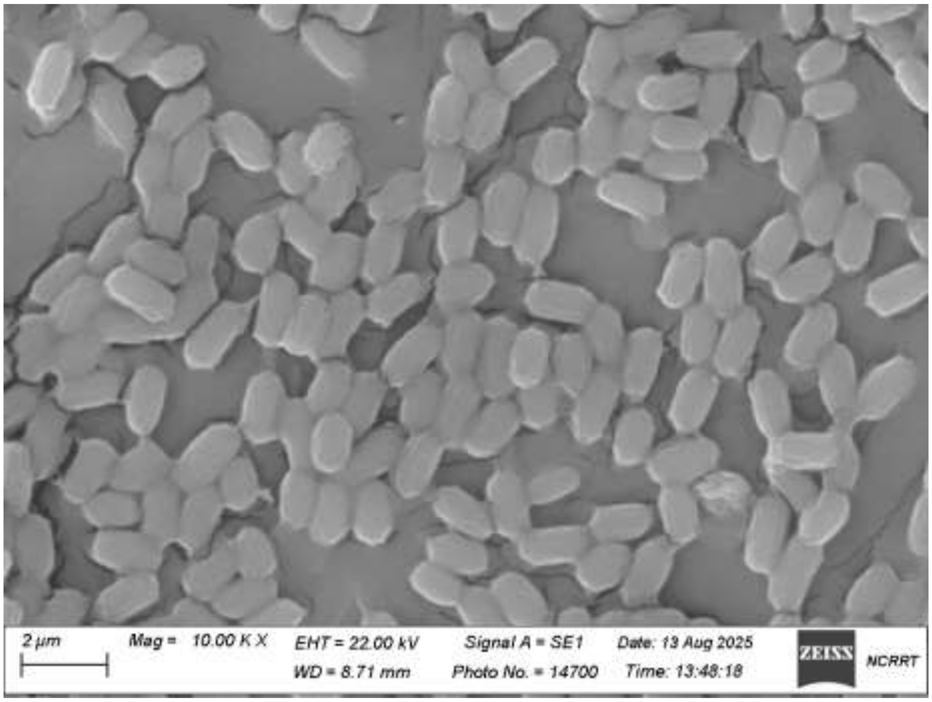
2 µm
Mag = 10.00 K X
EHT = 22.00 kV
WD = 8.71 mm
Signal A = SE1
Photo No. = 14700
Date: 13 Aug 2025
Time: 13:48:18
ZEISS
NCRRT

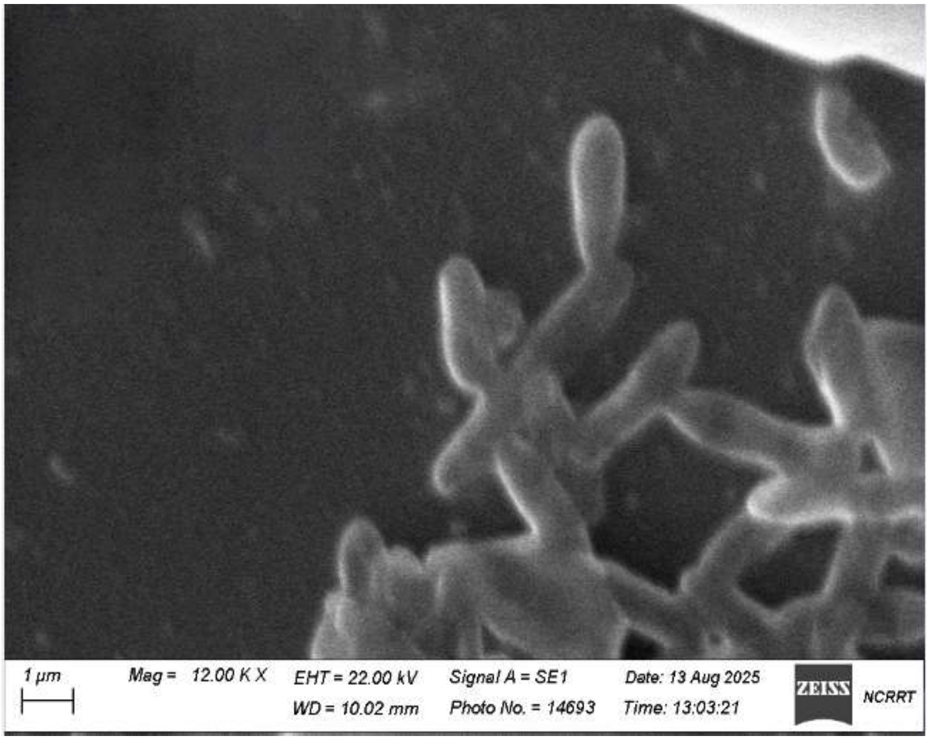
1 µm
Mag = 12.00 K X
EHT = 22.00 kV
WD = 10.02 mm
Signal A = SE1
Photo No. = 14693
Date: 13 Aug 2025
Time: 13:03:21
ZEISS
NCRRT

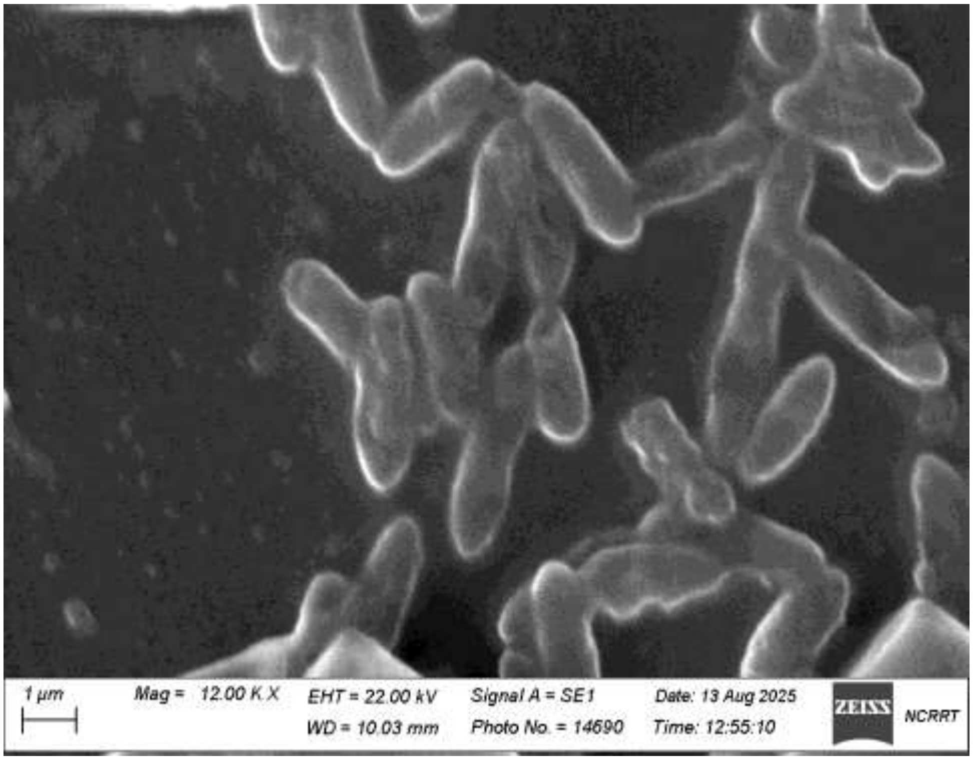

Fig. 6 SEM images showing inhibition of biofilm formation by *P. aeruginosa* PAO1. (A) Untreated PAO1 control on glass coverslips; (B) glass coverslips treated with B4 CFS; (C) glass coverslips treated with B4 PP-lactonase. Images captured at 12,000× magnification
